# Bicyclo[1.1.0]tetragermane‐2,4‐diide Diradicaloid

**DOI:** 10.1002/anie.202513772

**Published:** 2025-08-07

**Authors:** Falk Ebeler, Yury V. Vishnevskiy, Jan‐Hendrik Lamm, Beate Neumann, Hans‐Georg Stammler, Rajendra S. Ghadwal

**Affiliations:** ^1^ Molecular Inorganic Chemistry and Catalysis, Inorganic and Structural Chemistry, Center for Molecular Materials, Faculty of Chemistry Universität Bielefeld Universitätsstrasse 25 D‐33615 Bielefeld Germany

**Keywords:** Carbene, Cluster, Diradicaloid, Germanium, Main group, Stretched bond

## Abstract

*The synthesis, structure, and reactivity of the bicyclo[1.1.0]tetragermane‐2,4‐diide compound [(ADC)Ge_2_]_2_ (* ***3*** *), which features a Ge_4_ core bridged by two anionic dicarbene frameworks (ADC = PhC{N(Dipp)C}_2_; Dipp = 2,6‐iPr_2_C_6_H_3_), are reported. Treatment of an alkyne‐functionalized amidine Me_3_SiC≡CN(Dipp)C(Ph)═N(Dipp) (* ***1*** *) with GeCl_4_ affords [(ADC)GeCl_3_(GeCl_4_)] (* ***2*** *). KC_8_ reduction of* ***2*** *yields* ***3*** *as a Venetian red crystalline solid. DFT calculations reveal a singlet ground state for* ***3*** *with the singlet‐triplet energy gap of 14 kcal mol^−1^. CASSCF (complete active space self‐consistent field) calculations suggest a modest diradical character (β = 9%) for* ***3*** *. Compound* ***3*** *readily reacts with TEMPO (2,2,6,6‐tetramethylpiperidinyloxyl) to yield the Ge─Ge bond‐cleaved product, [(ADC)Ge(Ge‐TEMPO)]_2_ (* ***4*** *). Treatment of* ***3*** *with Fe_2_(CO)_9_ gives [(ADC)Ge(Ge{Fe(CO)_4_})]_2_ (* ***5*** *)*.

## Graphical abstract

The bicyclo[1.1.0]tetragermane‐2,4‐diide diradicaloid compound [(ADC)_2_Ge_4_] featuring a stretched bridgehead Ge*─*Ge bond is reported as a Venetian red crystalline solid. [(ADC)_2_Ge_4_] has been characterized by experimental and computational methods and its reactivity with TEMPO and Fe_2_(CO)_9_ has been explored.

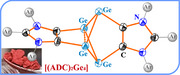

## Introduction

**Figure 1:**
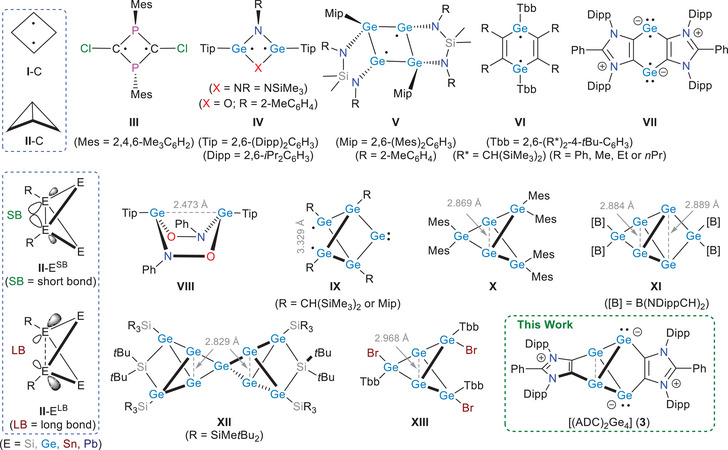
Schematic illustration of cyclobutanediyls **I**‐C, bicyclo[1.1.0]butanes **II**‐C, and their heavier Group 14 congeners **II**‐E^SB^ and **II**‐E^LB^, featuring a bridgehead short‐bond (SB) and long‐bond (LB), respectively (substituents omitted except R for **II**‐E). Niecke's diradicaloid **III** and selected germanium diradicaloids **IV**–**VII**. Representative examples of germanium diradicaloid (propellane‐type) cluster compounds **IX**–**XIII**.

Diradicals are molecules possessing two unpaired electrons in two (nearly) degenerate orbitals and may have either singlet or triplet electronic structures.^[^ ^1^, ^2^, ^3^ ^]^ Singlet diradicals (or diradicaloids) constitute a distinct class of open‐shell species that have attracted considerable attention as versatile building blocks for advanced functional materials in optoelectronic, spintronic, and energy‐related applications.^[^ ^4^, ^5^, ^6^, ^7^, ^8^, ^9^, ^10^, ^11^, ^12^, ^13^ ^]^ Furthermore, diradicals serve as key intermediates in various bond‐forming and bond‐cleavage reactions.^[^ ^14^, ^15^, ^16^ ^]^ Cyclobutanediyls (**I**‐C, Figure 1) represent prototypical examples of localized diradicals,^[^ ^17^ ^]^ exhibiting an exceptionally small singlet–triplet energy gap (Δ*E*_ST_ = +1.7 kcal mol^−1^).^[^ ^18^ ^]^ The radical recombination in **I**‐C to yield the corresponding bicyclo[1.1.0]butane (BCB) **II**‐C is thermodynamically favorable by 40–50 kcal mol^−1^ (for H substituents), however, the transformation is spin‐forbidden.^[^ ^19^, ^20^ ^]^ Consequently, the triplet ground state of **I**‐C can be observed via electron paramagnetic resonance (EPR) spectroscopy.^[^ ^21^, ^22^ ^]^ The isolation of the first cyclic C_2_P_2_ singlet diradical **III**, a phosphorus analogue of **I**‐C, by Niecke et al. in 1995 constituted a landmark in the chemistry of stable main‐group diradicals.^[^ ^23^ ^]^ This breakthrough spurred further progress in the field, leading to the isolation of several four‐membered cyclic singlet diradical(oid) systems.^[^ ^24^ ^]^

Among germanium compounds, Power et al. reported the cyclic diradicaloids **IV** (Figure 1).^[^ ^25^, ^26^ ^]^ In 2024, Wang, Wu, and colleagues described the synthesis of a cyclic singlet diradical **V**, an all‐germanium analogue of **I**‐C, with the calculated Δ*E*_ST_ of 22.9 kcal mol^−1^ (at B3LYP).^[^ ^27^ ^]^ In 2019, Sasamori and co‐workers reported stable 1,4‐digermabenzenes **VI** featuring three‐coordinated germanium atoms.^[^ ^28^ ^]^ In 2021, Ghadwal and co‐workers isolated an annulated 1,4‐digermabenzene‐1,4‐diide **VII** containing formally Ge(I) atoms in a two‐coordinated environment.^[^ ^29^ ^]^ Both **VI** and **VII** exhibit significant diradical character and readily cleave dihydrogen at room temperature.^[^ ^28^, ^29^ ^]^ Power et al. also reported a bicyclo[2.2.0]hexane diradicaloid **VIII** with a strained Ge─Ge bond (2.4731(7) Å), which reacts further with PhNO to form the corresponding Ge(IV) compound.^[^ ^30^ ^]^

BCBs (**II**‐C), the smallest and most strained saturated bicycles (∼64 kcal mol^−1^ for H substituents), serve as prototypes for various polycyclic compounds, including higher propellanes.^[^ ^31^ ^]^ They exhibit a promising reactivity profile for the discovery of new organic molecules and materials.^[^ ^32^, ^33^, ^34^, ^35^ ^]^ Among their heavier Group 14 analogues **II**‐E (Figure 1, substituents omitted except R on the bridgehead atoms),^[^ ^36^, ^37^ ^]^ silicon and germanium‐containing derivatives have been widely explored through theoretical studies.^[^ ^38^, ^39^, ^40^, ^41^, ^42^, ^43^ ^]^ A key structural feature of **II**‐E is the bond‐stretch isomerism,^[^ ^44^ ^]^ giving rise to short‐bond (**II**‐E^SB^) and long‐bond (**II**‐E^LB^) isomers. These isomers mainly differ in the length of the bridgehead bonds, which arise by the overlap of the high p‐character orbitals on each bridgehead atom.^[^ ^45^, ^46^, ^47^ ^]^ In **II**‐E^SB^, the bridgehead bond is considered as a classical covalent bond, while in **II**‐E^LB^, it is classified as a nonclassical singlet diradicaloid.^[^ ^48^, ^49^ ^]^ Since the isolation of the first bicyclo[1.1.0]tetrasilane species by Masamune and co‐workers in 1985,^[^ ^50^, ^51^ ^]^ several BCB‐derivatives containing silicon/germanium (i.e., mixed) have been reported.^[^ ^36^, ^37^, ^45^, ^46^, ^47^ ^]^ However, no stable example of bicyclo[1.1.0]tetragermane and bicyclo[1.1.0]tetrastannane molecules is known to date.^[^ ^45^, ^46^, ^47^ ^]^

Power and colleagues reported diradicaloids **IX**,^[^ ^52^, ^53^ ^]^ in which the elusive bicyclo[1.1.0]tetragermane moiety (**II**‐Ge) is capped by a germanium atom to result in Ge_5_‐clusters. In 2009, Breher et al. isolated the first pentagerma[1.1.1]propellane **X** in 4% yield,^[^ ^54^ ^]^ where the bridgehead germanium atoms of a bicyclo[1.1.0]tetragermane framework are connected via a Mes_2_Ge unit. Compound **X** exhibits 10% diradical character and radical‐type reactivity. Additional examples of molecules featuring stretched Ge─Ge bond(s) (2.829–2.968 Å) include **XI**,^[^ ^55^ ^]^ **XII**,^[^ ^56^ ^]^ and **XIII**,^[^ ^57^ ^]^ which are essentially derived by incorporating a silylene (R_2_Si) or germylene (R_2_Ge) unit into the Ge_4_‐scaffold (**II**‐Ge). Nevertheless, the isolation of a discrete bicyclo[1.1.0]tetragermane compound remains a formidable challenge to date.

Herein, we report the synthesis of bicyclo[1.1.0]tetragermane‐2,4‐diide compound **3**, in which the Ge_4_ framework is embedded between two anionic dicarbene (ADC) units,^[^ ^58^, ^59^, ^60^, ^61^, ^62^, ^63^, ^64^, ^65^ ^]^ and describe its (electronic) structure and reactivity.

## Results and Discussion

**Figure 2:**
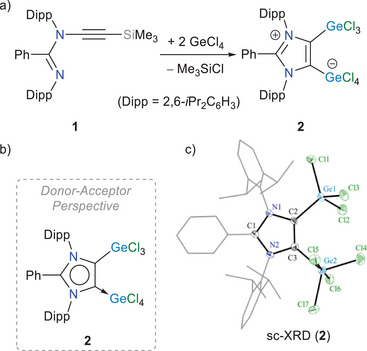
a) Synthesis of compound **2**. b) Donor–acceptor perspective for **2**. c) Solid‐state molecular structure of **2**. Aryl groups are shown as wireframes and H atoms are omitted for clarity. Thermal ellipsoids are shown at 50%. Selected bond lengths (Å) and angles (°): C2─C3 1.373(4), Ge1─C2 1.944(3), Ge2─C3 1.995(3), Ge1─Cl1 2.112(1), Ge1─Cl3 2.117(1), Ge2─Cl4 2.164(1), Ge2─Cl5 2.337(1); C3─C2─Ge1 129.0(2), C2─C3─Ge2 130.7(2), C2─Ge1─Cl1 116.1(1), C3─Ge2─Cl4 124.2(1), C3─Ge2─Cl5 85.0(1), Cl5─Ge2─Cl6 177.3(1).

Treatment of the alkyne functionalized amidine **1** (Figure 2a), the ring‐opened form of a mesoionic carbene (iMIC),^[^ ^66^ ^]^ with two equivalents of GeCl_4_ in toluene afforded the ring‐closing product **2** as an off‐white solid in 80% yield. The only byproduct, Me_3_SiCl, was easily removed by washing with toluene. Compound **2** can be described either as a Cl_3_Ge‐functionalized iMIC adduct of GeCl_4_ (see the donor–acceptor depiction in the Figure 2b)^[^ ^67^, ^68^ ^]^ or as an anionic dicarbene (ADC) compound [(ADC)GeCl_3_(GeCl_4_)], where ADC = PhC{N(Dipp)C}_2_ and Dipp = 2,6‐*i*Pr_2_C_6_H_3_. The ^1^H and ^13^C NMR spectra of **2** display the expected signals for the ADC moieties (see Supporting Information).^[^ ^67^, ^68^, ^69^, ^70^, ^71^, ^72^, ^73^, ^74^ ^]^ The ^1^H NMR spectrum shows two doublets and one septet characteristic for the isopropyl groups. The solid‐state molecular structure of **2**, determined by single crystal X‐ray diffraction (sc‐XRD) (Figure 2c), shows the expected atom connectivity.^[^ ^75^ ^]^ The Ge2─C3 bond length (1.995(3) Å) and Ge2─Cl4/Cl5 bond lengths (2.164(1)/2.337(1) Å) are consistent with those observed in (NHC)GeCl_4_compounds (NHC = *N*‐heterocyclic carbene), where C_NHC_─Ge bonds measured around 1.992 Å and Ge─Cl bond lengths range from 2.143 to 2.320 Å, characteristic for five coordinated germanium centers.^[^ ^76^, ^77^ ^]^ Similarly, the Ge1─C2 bond length (1.944(3) Å) and the average Ge1─Cl distance (∼2.112 Å) are comparable with those of four‐coordinated Ge(IV) chlorides, which typically exhibit Ge─C_Ar_ bond lengths of ∼1.92 Å and Ge─Cl bond lengths of 2.16 Å.^[^ ^78^ ^]^

**Scheme 1:**
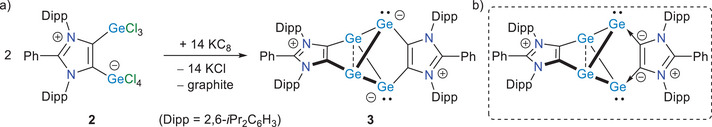
a) Synthesis of compound [(ADC)Ge_2_]_2_ (**3**). b) An alternative canonical form of **3**.

Treatment of a THF solution of compound **2** with seven equivalents of KC_8_ produced the Ge_4_‐cluster compound [(ADC)Ge_2_]_2_ (**3**) as a Venetian red solid in 97% yield (Scheme 1a). The ^1^H and ^13^C{^1^H} NMR spectra of **3** show the expected signals for the ADC units, which differ from those observed in **VII** (Figure 1) featuring a planar tricyclic ring system.^[^ ^29^ ^]^ The ^1^H NMR spectrum reveals two septets and four doublets corresponding to the isopropyl group, along with characteristic signals for the aryl and imidazole moieties.

**Figure 3:**
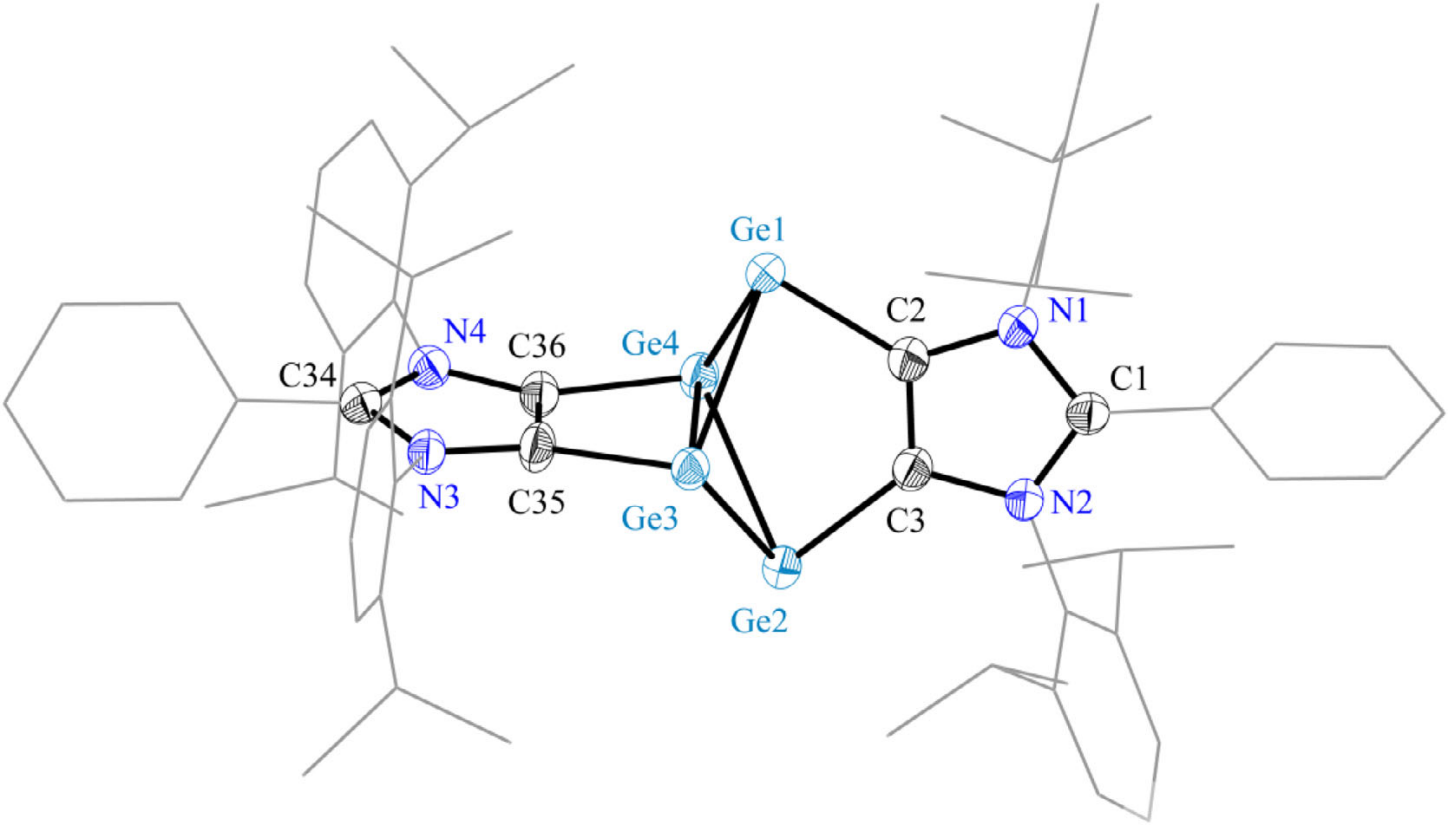
Solid‐state molecular structure of **3**. Aryl groups are shown as wireframe models. H atoms and minor occupied disordered atoms as well as solvent molecules are omitted for clarity. Ellipsoids are shown at 50% (Selected bond lengths and angles are given in Table 1).

The solid‐state molecular structure of **3** (Figure 3) reveals a central Ge_4_ core adopting a bicyclo[1.1.0]tetragermane motif embedded by two ADC units. As in a hypothetical species **II**‐Ge, the bridgehead germanium atoms (Ge3 and Ge4) are four‐coordinated, while Ge1 and Ge2 are three‐coordinated, each retaining a lone pair. This may loosely be illustrated by the Lewis structure (Scheme 1b), where one imidazole unit acts as a chelating dianionic substituent for the Ge3/Ge4 atoms, while the second imidazole unit functions as a bis‐carbene Lewis base coordinating the Lewis‐acidic (divalent) Ge1/Ge2 centers (see Natural Bond Orbital (NBO) analyses below). The Ge─C bond lengths (1.996(3)–2.067(3) Å) are only marginally larger than those in the previously reported Ge(I) diradicaloid **VII** with an annulated C_4_Ge_2_‐ring (1.963(2) and 1.960(2) Å).^[^ ^29^ ^]^ The central Ge_4_ unit of **3** exhibits positional disorder, with the C2─C3 and Ge1─Ge2 connections (68%) partially interchanged with Ge3─Ge4 (32%). The peripheral 1,3‐imidazole units form a dihedral angle of 79.5°, consistent with their orthogonal bridging of the Ge_4_ core. The values of Ge1─Ge3 (2.517(2) Å), Ge1─Ge4 (2.564(2) Å), Ge2─Ge3 (2.572(1) Å), and Ge2─Ge4 (2.514(1) Å) bond lengths closely match those reported for Wang's tetragermacyclobutane‐1,3‐diyl **V** (2.511(1) and 2.548(1) Å).^[^ ^27^ ^]^ They are also comparable to that of a 1,3‐digermabicyclo[1.1.0]butane featuring an inverted bridge Ge─Ge bond (2.583(1) Å).^[^ ^79^ ^]^ However, they are slightly longer than the corresponding distances in the Ge_6_‐cluster **XI** (2.490(1) and 2.468(1) Å)^[^ ^55^ ^]^ and in cyclotetragermanes (2.45–2.49 Å).^[^ ^80^, ^81^ ^]^ Notably, the interatomic distance between the bridgehead Ge3 and Ge4 atoms is 2.752(4) Å that is significantly longer than the sum of the covalent single‐bond radii of two germanium atoms (2.42 Å),^[^ ^82^ ^]^ yet shorter than their combined van der Waals radii (4.22 Å).^[^ ^83^ ^]^ This interatomic separation of 2.752(4) Å in compound **3** is reasonably close to that observed in the diradicaloid **X** (2.869(2) Å)^[^ ^54^ ^]^ and the tetraradicaloid **XII** (2.829(1) Å),^[^ ^56^ ^]^ suggesting a possible diradicaloid character.

To obtain further insight into the electronic structures of **3**, we performed DFT calculations (see Supporting Information). The optimized molecular structure of **3** (Figure S27) for a closed‐shell singlet (**CS**) solution at the PBEh‐3c level of theory^[^ ^84^ ^]^ is in good qualitative agreement with its sc‐XRD structure (Figure 3 and Table 1). Nonetheless, the triplet (**T**) solution for **3** is calculated to be only 14 kcal mol^−1^ higher in energy, which is considerably lower than that calculated for Wang's diradicaloid **V** (22.9 kcal mol^−1^).^[^ ^27^ ^]^ The NBO findings (see Table 1 for natural charges and Wiberg bond indices, WBIs) are in line with sc‐XRD data of **3**. A considerably smaller value of the WBI for the Ge3─Ge4 bond (0.57) than those of Ge1/Ge2─Ge3/Ge4 bonds (∼0.77) suggests a weaker interaction between the bridgehead germanium atoms. These values of WBIs for Ge─Ge bonds in **3** (0.57 and 0.77) are comparable to the corresponding bonds in Breher's diradicaloid **X** (0.55 and 0.81).^[^ ^54^ ^]^ The WBI for the Ge1⋯Ge2 pair amounts to 0.17, excluding any bonding interactions between the Ge1 and Ge2 atoms (see below).

**Table 1:** Selected sc‐XRD [calculated at PBEh‐3c] bond lengths [Å] and angles [°] of **3**. Calculated natural charges (*q*) and Wiberg bond indices (WBIs) for **3**.

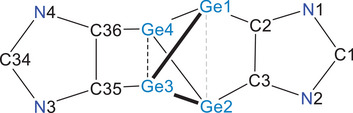		Bond	WBI	atom	charge (*q*)
		Ge1⋯Ge2	0.17	Ge1/Ge2	0.10
		Ge1─Ge3	0.77	Ge3/Ge4	0.15
		Ge1─Ge4	0.76	C2/C3	−0.24
		Ge3─Ge4	0.57	C36/C35	−0.21
		Ge1─C2	0.75	N1/N2	−0.37
		Ge3─C35	0.69	N3/N4	−0.34
Bond	sc‐XRD [calc.]	Angle		sc‐XRD [calc.]	
Ge1⋯Ge2	3.828(4) [3.832]	Ge1─Ge4─Ge2		97.8(1) [98.1]	
Ge1─Ge3	2.517(2) [2.527]	Ge1─Ge4─Ge3		56.4(1) [56.9]	
Ge1─Ge4	2.564(2) [2.546]	Ge1─Ge3─Ge4		58.0(1) [57.6]	
Ge3─Ge4	2.752(4) [2.747]	Ge4─Ge1─Ge3		65.6(1) [65.6]	
Ge1─C2	2.049(4) [2.024]	Ge3─Ge4─C36		69.4(1) [69.9]	

**Figure 4:**
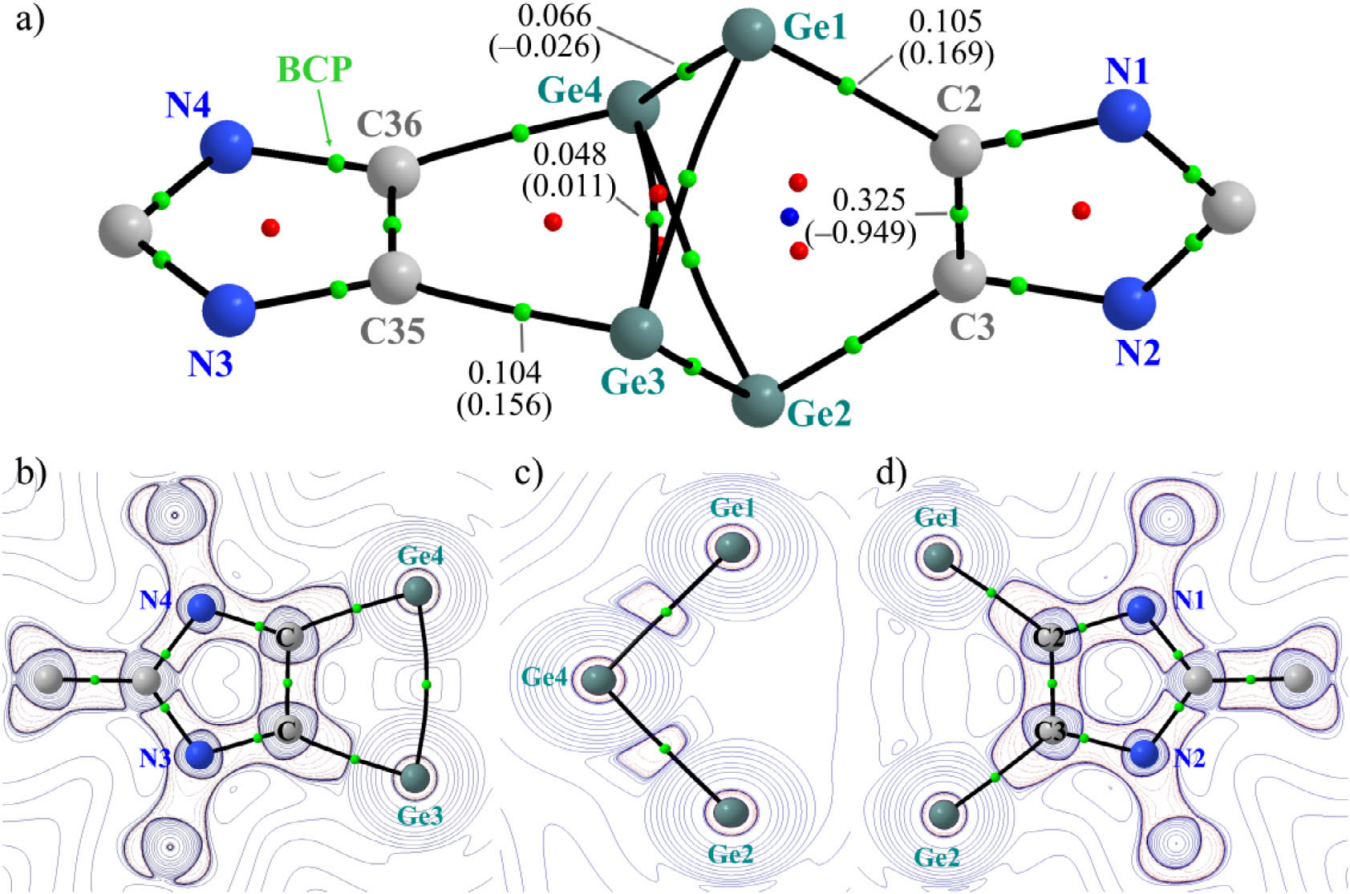
a) Molecular graph of **3** obtained from QTAIM analysis (PBE0/def2‐TZVPP). The small green, red, and blue spheres indicate BCPs (bond critical points), RCPs (ring critical points), and CCP (cage critical point), with electron density (*ρ*(r_BCP_) in a. u.) and Laplacian of electron density (∇^2^ *ρ*(r) in a. u.) (in parentheses). Contour plots of ∇^2^ *ρ*(r) (solid lines correspond to positive values and dashed to negative values) for the b) Ge3─C35─C36─Ge4 c) Ge1─Ge4─Ge2, and (d) Ge1─C2─C3─Ge2 planes.

The **CS** singlet wavefunction of **3** (at PBE0/def2‐TZVPP) was further examined by topological analysis according to Bader's quantum theory of atoms‐in‐molecules (QTAIM).^[^ ^85^ ^]^ The characteristics of a bond can be comprehended by evaluating the electron density (*ρ*(**r**_BCP_)) and its derivatives at the bond critical point (BCP).^[^ ^86^, ^87^, ^88^, ^89^ ^]^ The Ge1─Ge3/Ge4, Ge2─Ge3/Ge4, and Ge3─Ge4 bonds in **3** (Figure 4) are confirmed by BCPs, while no BCP is present between Ge1 and Ge2 atoms. The electron density at the critical points for all Ge─C bonds in **3** is nearly identical (0.104, 0.105 a. u.), but the same at the Ge1─Ge3 (0.066 a. u.) and Ge3─Ge4 bonds (0.048 a. u.) is distinct (Figure 4a). The Laplacian of electron density (∇^2^ *ρ*(**r**_BCP_)) at the BCP of Ge3─Ge4 bond (0.011 a. u.) is smaller and positive, while the same for Ge1/Ge2─Ge3/Ge4 bonds (−0.026 a. u.) is rather large and negative (Figure 4a). The negative value of ∇^2^ *ρ*(**r**), which resides mainly in the charge accumulation region of Ge1/Ge2─Ge3/Ge4 bonds (Figure 4c), may be roughly interpreted as an indicator of covalent bonding. Notably, beside the small positive value of ∇^2^ *ρ*(**r**) for the Ge3─Ge4 bond, no charge accumulation is visible at the BCP (Figure 4b), indicating a weak interaction. This feature is in line with other propellane derivatives and related Group 14 compounds.^[^ ^90^, ^91^, ^92^ ^]^ The positive values (∼0.16 a. u.) of ∇^2^ *ρ*(**r**), which is located toward the Ge atom (Figure 4b,d), for the Ge─C bonds suggest a polar covalent bonding between germanium and carbon atoms.

**Figure 5:**
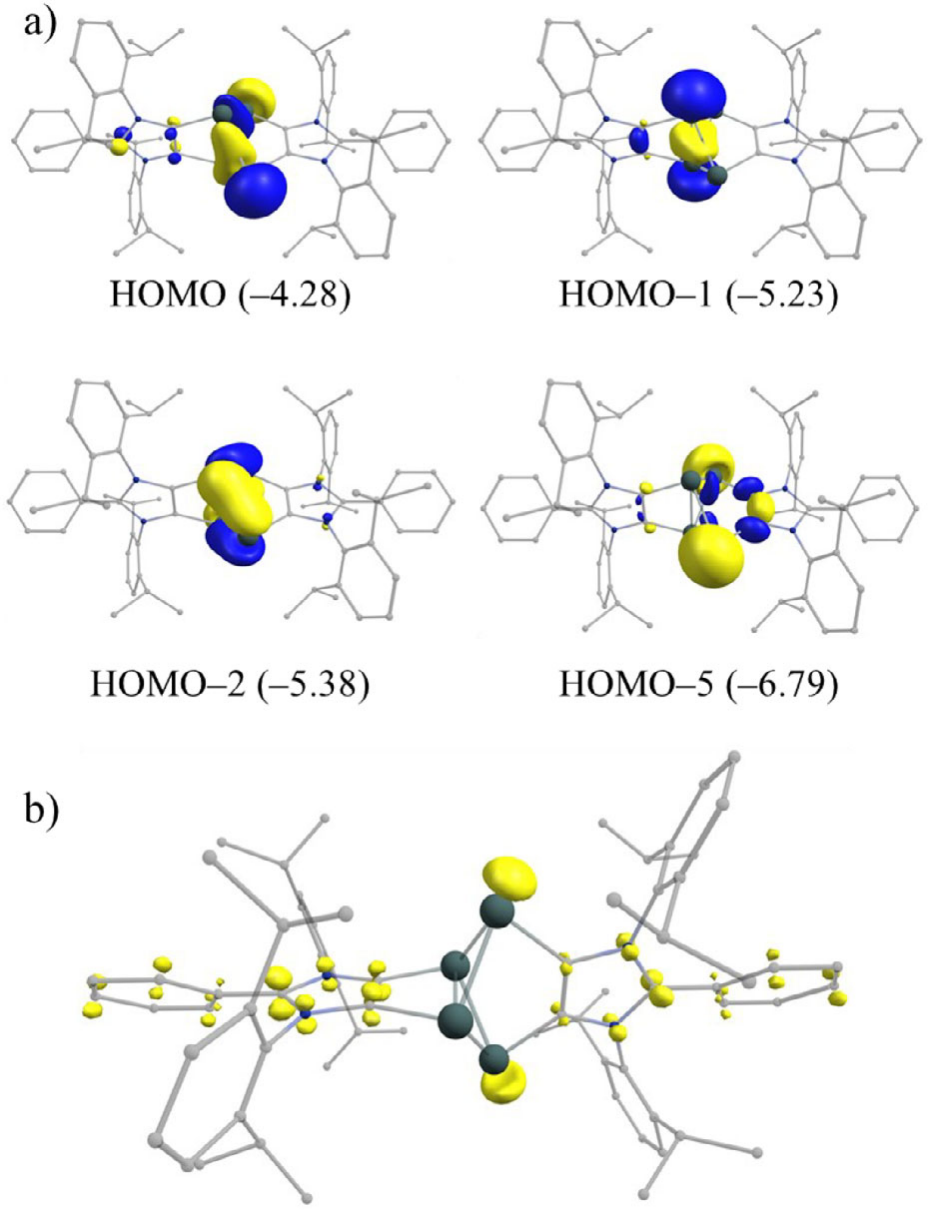
a) Selected molecular orbitals (with energies in eV at PBE0/def2‐TZVPP) for **3**. b) FOD plot (isosurfaces 0.007 a.e. in yellow) for **3**.

In compound **3**, the HOMO–1 is primarily the Ge3─Ge4 bonding orbital, whereas the HOMO involves both Ge_4_‐cluster bonding interactions and a combination of lone‐pair orbitals on the Ge1 and Ge2 atoms (Figure 5a). The HOMO–2 predominantly represents Ge_4_‐cluster bonding, while the HOMO–5 mainly corresponds to a second lone‐pair combination on Ge1 and Ge2, with some contribution from the ligand. The UV–vis spectrum of compound **3** (Figures S13 and S14) shows three main absorption bands at *λ*_max_ = 272, 321, and 551 nm. According to TD‐DFT calculations (Table S9), the band at 551 nm may be assigned to the HOMO→LUMO+1 transition. To further investigate the electronic structure of **3**, we also executed fractional occupation number weighted density (FOD) calculations^[^ ^93^ ^]^ as a diagnostic for static electron correlation (SEC) (Figure 5b). FOD analysis is a reliable method for identifying the localization of “hot” electrons (that are strongly correlated and chemically active) in a molecule. The calculated FOD number (*N*_FOD_) for **3** is 2.88e, indicating a moderate level of SEC. Notably, the FOD is relatively low and is primarily localized on the germanium lone pairs (Figure 5b).

**Figure 6:**
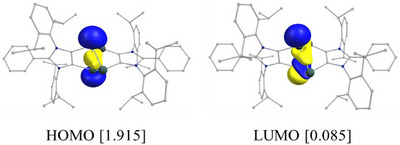
CAS(2,2) orbitals for **3** according to SS‐CASSCF calculations [occupation number].

To more rigorously assess the extent of diradical character in compound **3**, we performed complete active space self‐consistent field (CASSCF) calculations using a CAS(2,2) active space (see Supporting Information for detail). This minimal active space, which includes two electrons in two orbitals, was chosen to capture the essential open‐shell singlet features and to evaluate the multireference nature of the ground state. It specifically accounts for the key frontier orbitals involved in potential diradical configurations, providing a reliable estimate of diradical character. The CAS orbitals with their occupation numbers according to state specific (SS)‐CASCF calculations are shown in Figure 6. The HOMO of SS‐CASSCF calculations is a σ‐bonding orbital between the bridgehead germanium atoms, while the LUMO is the corresponding σ*‐orbital. The occupation numbers for these orbitals amount to 1.915 and 0.085. The calculated diradical character (*β*) for **3** amounts to 9%, which compares well with that of **X** (10%).^[^ ^54^ ^]^

**Scheme 2:**
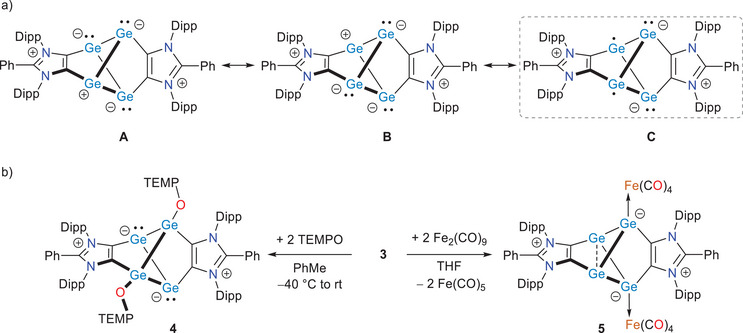
a) Selected canonical structures (**A**, **B**, and **C**) for **3**. b) Reactions of **3** with TEMPO and Fe_2_(CO)_9_ to **4** and **5**, respectively.

Having analyzed the electronic structure and identified a moderate diradical character in compound **3**, we were prompted to explore its chemical reactivity. The elongated bridgehead bond (Ge3─Ge4) likely underlines its radical‐like behavior. This can be rationalized by considering zwitterionic resonance structures **A** and **B** (Scheme 2a), which are characteristic of main‐group singlet diradicaloids and are conceptually equivalent to the diradical species **C**.^[^ ^1^, ^2^, ^3^ ^]^ In structure **C**, each of the four three‐coordinated germanium atoms has an equal probability of hosting either a lone pair or an unpaired electron. As a result, both radical and germylene‐type reactivity pathways are equally plausible at all germanium atoms. In fact, compound **3** readily reacts at room temperature with TEMPO (2,2,6,6‐tetramethylpiperidinyloxyl), a stable nitroxyl radical, to afford compound **4** as a dark green crystalline solid in 72% yield (Scheme 2b). In addition to the expected signals for the ADC units, the ^13^C{^1^H} NMR spectrum of **4** shows characteristic signals at 59.5 (*C*Me_2_) and 41.1 ppm (*C*H_2_) for the TEMPO moiety. The formation of **4** emphasizes the diradical reactivity of **3**, where the Ge1 and Ge3 atoms (cf. Figure 3 and canonical structure **C** in Scheme 2a) undergo radical combination with TEMPO to result in Ge─O bonds. Treatment of **3** with Fe_2_(CO)_9_ affords compound **5** as a brown solid. The ^1^H NMR spectrum of **5** exhibits a similar signal pattern as observed for **3** with somewhat downfield shifting. In addition to the expected signals for the ADC units, the ^13^C{^1^H} NMR spectrum of **5** features a signal at 217.1 ppm for the carbonyl groups. Moreover, the IR spectrum of **5** (Figures S21 and S22) contains absorption bands at 2008, 1933, 1915, and 1904 cm^−1^ for ν(CO) stretching vibrations, which are characteristic of metallylenes‐Fe(CO)_4_ complexes.^[^ ^94^, ^95^, ^96^ ^]^

**Figure 7:**
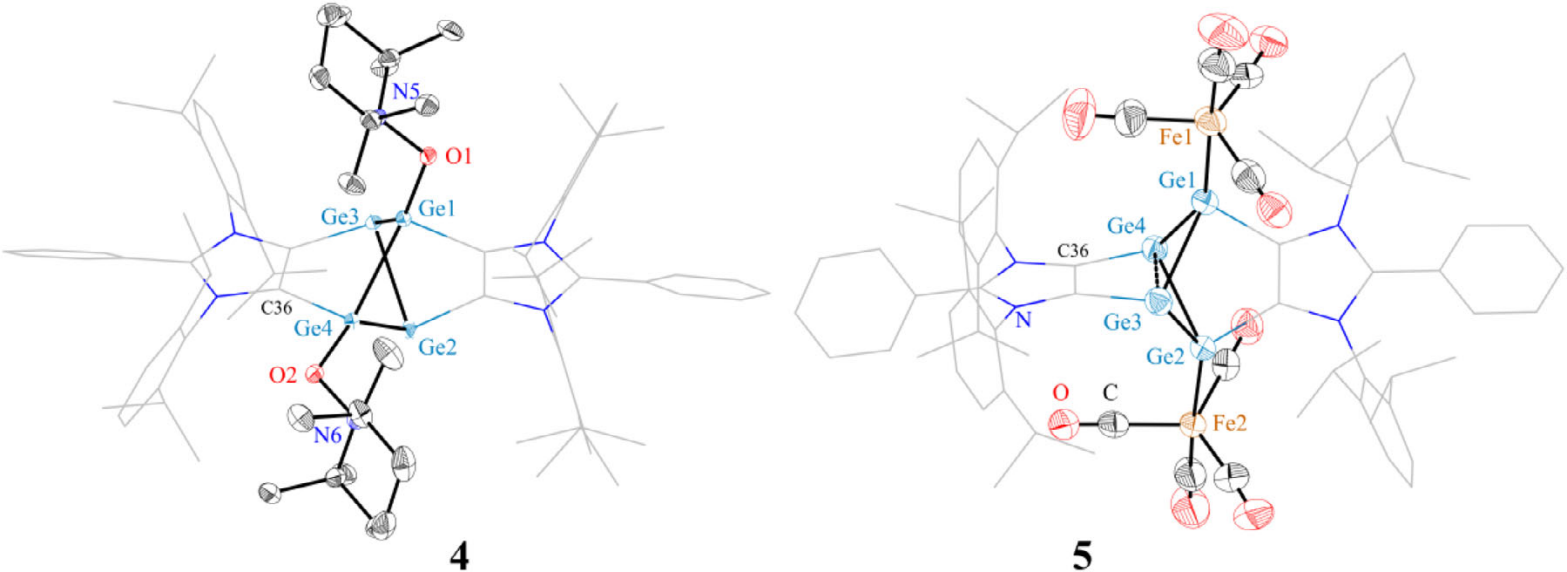
Solid‐state molecular structures **4** and **5**. Aryl groups are shown as wireframes and H atoms and occupied disordered atoms are omitted for clarity. Thermal ellipsoids are shown at 50%. Selected bond lengths (Å) and angles (°) for **4**: Ge1─O1 1.858(1), Ge1─Ge3 2.568(1), Ge2─Ge4 2.529(1), Ge1⋯Ge2 3.337(1), Ge3⋯Ge4 3.302(2); Ge1─Ge4─Ge2 82.4(1); for **5**: Ge1⋯Ge2 3.482(1), Ge1─Ge4 2.462(1), Ge1─Ge3 2.594(1), Ge3─Ge4 2.967(1), Ge1─Fe1 2.360(1), Ge2─Fe2 2.363(4); Ge1─Ge4─Ge2 87.4(1), Ge1─Ge4─Ge3 56.2(1), Ge1─Ge3─Ge4 52.0(1), Ge4─Ge1─Ge3 71.8(1), Ge3─Ge4─C36 65.7(2), Ge2─Ge4─Ge3─Ge1 117.3(1).

Compound **4** crystallizes with two independent molecules per asymmetric unit that differ only slightly in terms of their bond lengths and bond angles (see Supporting Information). The solid‐state molecular structure of **4** (Figure 7) reveals the presence of an ADC‐embedded Ge_4_‐moiety, incorporating two three‐coordinated and two four‐coordinated germanium atoms. Unlike **3**, in which both three‐coordinated germanium atoms are located on the same side, each of them is located on the opposite side. The transannular Ge⋯Ge distances (Ge1⋯Ge2: 3.337(1) Å and Ge3⋯Ge4: 3.302(2) Å) in **4** are comparable. These distances are similar to those of Power's Ge_5_‐cluster **IX** (3.329(1) Å)^[^ ^52^, ^53^ ^]^ as well as of Wang's tetragermacyclobutane‐1,3‐diyl **V** (3.254 Å).^[^ ^27^ ^]^ The Ge─O bond lengths of **4** (1.858(1)–1.864(1) Å) are in line with those of Kira's cyclic dialkylgermylene derived compound [R_2_Ge(TEMPO)_2_] (1.824(2), 1826(2) Å).^[^ ^97^ ^]^

The solid‐state molecular structure of **5** (Figure 7) shows that the bicyclo[1.1.0]tetragermane unit remains intact and each of the germanium atoms bearing a lone pair binds to the Fe(CO)_4_ Lewis acid fragment. The Ge1─Ge3 (2.594(1) Å) and Ge1─Ge4 (2.462(1) Å) bond lengths of **5** are comparable to those of **3** (2.517(2), 2.564(2) Å); however, the distance between bridgehead Ge3 and Ge4 atoms of **5** (2.967(1) Å) is larger (for **3**: 2.752(4) Å). Notably, the Ge3─Ge4 bond length of **5** (2.967(1) Å) compares well with that of **XIII** (2.968(1) Å).^[^ ^57^ ^]^ The distance between the Ge1 and Ge2 atoms of **5** (3.482(1) Å) is smaller than that of **3** (3.828(4) but is still larger than a stretched Ge─Ge single bond observed in **X–XIII** (2.829–2.883 Å).^[^ ^54^, ^55^, ^56^, ^57^ ^]^ The Ge1─Fe1 (2.360(2) Å) and Ge2─Fe2 (2.363(4) Å) bond lengths of **5** are comparable and agree well with those of known germylene‐Fe(0) complexes.^[^ ^98^, ^99^ ^]^

To shed light on the electronic structure of **5**, we performed DFT calculations at the PBEh‐3c as well as PBE0/def2‐TZVPP level of theory.^[^ ^84^ ^]^ The optimized molecular structure of **5** (Figure S37) is in good qualitative agreement with its sc‐XRD structure (Figure 7). The results of NBO analyses for **5** (Table S13) are in line with its sc‐XRD structure. The WBIs for Ge1─Ge3 (0.69) and Ge1─Ge4 (0.84) bonds are consistent with a classical Ge─Ge covalent bond, while the WBI for Ge3─Ge4 amounts to 0.48, suggesting a non‐classical (stretched) bond. We also performed a topological analysis of the electron density for **5** according to QTAIM using the similar wavefunction (PBE0/def2‐TZVPP). The molecular graph (Figure S38) and details of QTAIM findings for **5** (Table S14) are given in the Supporting Information. For **5**, the Ge1─Ge3, Ge1─Ge4, Ge2─Ge3, and Ge2─Ge4 bonds are confirmed by BCPs. However, no BCP for the Ge3─Ge4 bond, which (with the value 2.968 Å, Figure 7) still falls in the range of stretched Ge─Ge bonds for nonclassical diradicaloids (Figure 1), is present (Figure S39). This is likely due to the substantial electron density transfer to the coordinating Fe(CO)_4_ moieties in **5**. Moreover, like in **3**, no charge accumulation is evident at the center of Ge3 and Ge4 atoms of **5**.

## Conclusions

In conclusion, the bicyclo[1.1.0]tetragermane‐2,4‐diide compound **3** has been isolated as a Venetian red crystalline solid in 97% yield. As expected for a bicyclo[1.1.0]tetragermane species (cf. **II**‐Ge), compound **3** features a butterfly‐shaped Ge_4_ cluster bridged by two 1,3‐imidazole‐based anionic dicarbenes (ADCs). Structural and computational analyses indicate a modest diradical character (*β* = 9%), primarily attributed to the elongated bridgehead Ge─Ge bond, a characteristic of nonclassical diradicaloids. Reaction of **3** with TEMPO affords compound **4**, demonstrating diradical reactivity via cleavage of the bridgehead Ge─Ge bond. In contrast, treatment of **3** with Fe_2_(CO)_9_ yields compound **5**, in which the distance between the bridgehead Ge atoms further stretched to 2.967(1) Å (cf. 2.752(4) Å for **3**), reflecting germylene‐type reactivity of **3**. In **5**, the coordination of Fe(CO)_4_ fragments to the germanium atoms, each bearing a lone pair, further elongates the bridgehead Ge─Ge bond compared to that in **3**, suggesting an increase in diradical character upon metal coordination. These findings highlight the potential of compound **3** to advance fundamental studies in main group and diradical chemistry. Future research may focus on developing new open‐shell main group species (e.g., neutral or charged radicals) and organometallic compounds with potential applications in synthesis and catalysis.

## Supporting Information

Experimental details, the plots of NMR, IR, and UV‐vis spectra as well as the detail of single‐crystal X‐ray crystallography and quantum chemical calculations of the reported compounds are given in the Supporting Information.

## Conflict of Interests

The authors declare no conflict of interest.

## Supporting information



Supporting Information

Supporting Information

## Data Availability

The data that support the findings of this study are available in the Supporting Information of this article.
