# Supplementary material for: Bicyclo[1.1.0]tetragermane‐2,4‐diide Diradicaloid
Source: Angew Chem Int Ed Engl. 2025 Aug 7;64(38):e202513772. doi: 10.1002/anie.202513772 (PMC12435412; doi:10.1002/anie.202513772)
Supplement: Supplementary file 1 — Supporting Information [file ANIE-64-e202513772-s001.pdf]

Supporting Information

for

Bicyclo[1.1.0]tetragermane-2,4-diide Diradicaloid

Falk Ebeler, Yury V. Vishnevskiy, Jan-Hendrik Lamm, Beate Neumann, Hans-Georg Stammler and Rajendra S. Ghadwal*

Molecular Inorganic Chemistry and Catalysis, Inorganic and Structural Chemistry, Center for Molecular Materials, Faculty of Chemistry, Universität Bielefeld, Universitätsstrasse 25, D-33615, Bielefeld, Germany.

**E-Mail: rghadwal@uni-bielefeld.de; http://www.ghadwalgroup.de*

Fax: +49 521 106 6026; Tel: +49 521 106 6167

Table of Contents

Experimental Section **S1**

Synthesis of [{(ADC)GeCl$_3$}GeCl$_4$] (**2**) S1

Synthesis of [(ADC)Ge$_2$]$_2$ (**3**) S2

Synthesis of [(ADC)Ge{Ge(TEMPO)}]$_2$ (**4**) S2

Synthesis of [(ADC)Ge(Ge{Fe(CO)$_4$})]$_2$ (**5**) S3

Plots of the NMR-Spectra **S5**

Plots UV-Vis Spectra **S11**

Plots of Infrared Spectra **S14**

Crystallographic Details **S16**

Molecular Structures S19

Quantum Chemical Calculations **S21**

References **S41**

Experimental Section

All experiments and manipulations were carried out under an inert gas atmosphere of argon or nitrogen using standard Schlenk techniques or an MBraun LABmaster Pro glovebox. Organic solvents [THF (tetrahydrofuran), Me-THF, toluene (PhMe), benzene (C_6H_6), *n*-hexane and *n*-heptane (over NaK); fluorobenzene (PhF) (over CaH_2)] were dried by refluxing over an appropriate drying agent, distilled prior to use, and stored over 4 Å molecular sieve. Deuterated solvents were dried over appropriate drying agents, distilled, and stored inside a glove box. The alkyne functionalized amidine compound **1** was prepared as described previously by this laboratory.[1] NMR spectra were recorded on a Bruker Avance III 500, a Bruker Avance III 500 HD or a Bruker Avance III 600 spectrometer. Chemical shifts are given in δ ppm (br = broad, d = doublet, t = triplet; *p*t = *pseudo*-triplet, sept = septet, m = multiplet) and were referenced to the solvent residual signal: $CDCl_3$ (^{1}H 7.26, ^{13}C 77.16 ppm); THF-d_8 (^{1}H 3.58, ^{13}C 67.21 ppm); C_6D_6 (^{1}H 7.16, ^{13}C 128.06).[2] Infrared (IR) spectra were recorded on a Bruker Alpha FTIR spectrometer equipped with an ATR module at room temperature (rt) under nitrogen atmosphere with 32 scans. Melting points (MPs) were measured using a Büchi B-545 melting point apparatus. Elemental analyses were carried out with a EURO EA Element Analyzer. UV-Vis spectra were recorded on a Thermos Scientific Genesys 50 spectrophotometer. *n*-BuLi (2.5 M in hexanes, Sigma Aldrich) were used as supplied. $GeCl_4$ and Me_3SiCl (Fisher Scientific) were freshly distilled before use. The term "ADC" (anionic dicarbene) is used for the cyclic framework PhC[N(Dipp)C]$_2$ (Dipp = 2,6-*i*$Pr_2C_6H_3$), which serve as a mono-anionic four electron donor (*i.e.* "LX" type ligand).

Synthesis of [{(ADC)$GeCl_3$}$GeCl_4$] (2)

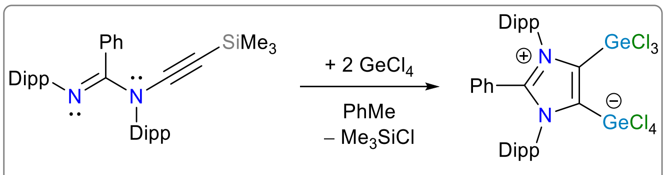

To a 10 mL toluene solution of **1** (3.22 g, 6.00 mmol) was added $GeCl_4$ (1.60 mL, 14.40 mmol) at room temperature (rt) and the resulting dark brown suspension was stirred for 12h. The precipitate was separated by filtration, washed with toluene (3x10 mL), and dried under vacuum to obtain compound [{(ADC)$GeCl_3$}$GeCl_4$] (**2**) as an off-white solid in 80% (4.12 g, 4.81 mmol) yield. Crystals suitable for single crystals X-ray diffraction (sc-XRD) were obtained by storing a saturated toluene solution of **2** at –24 °C for one week. **MP**: °C 210 (dec.). Elemental analysis (%) calculated for $C_{33}H_{39}Cl_7Ge_2N_2$ (857.1): C 46.24, H 4.59, N 3.27; found: C 45.98, H 4.62, N 3.20. **^{1}H NMR** (500 MHz, THF-d_8, 298 K): 7.67 (t, J = 7.9 Hz, 2H, *p*-C_6H_3), 7.45 (d, J = 7.8 Hz, 4H, *m*-C_6H_3), 7.38 (t, J = 7.6 Hz, 1H, C_6H_5), 7.13 (t, J = 7.5 Hz, 2H, C_6H_5), 6.82 (d, J = 7.6 Hz, 2H, C_6H_5), 2.94 (sept, J = 6.7 Hz, 4H, C*H*(CH_3)$_2$), 1.38 (d, J =

6.6 Hz, 12H, CH(C*H*$_3$)), 0.71 (d, *J* = 6.8 Hz, 12H, CH(C*H*$_3$)) ppm. **^{13}C{^{1}H} NMR** (126 MHz, THF-*d*$_8$, 298 K): 147.0 (Ge*C*), 146.3, 134.0, 133.9, 132.0, 131.5, 129.8, 127.2, 121.7 (*C*$_6$H$_3$, *C*$_6$H$_5$, N*C*N), 29.9, (*C*H(CH$_3$)$_2$), 25.2, 23.9 (CH(*C*H$_3$)$_2$) ppm.

Synthesis of [(ADC)Ge$_2$]$_2$ (3)

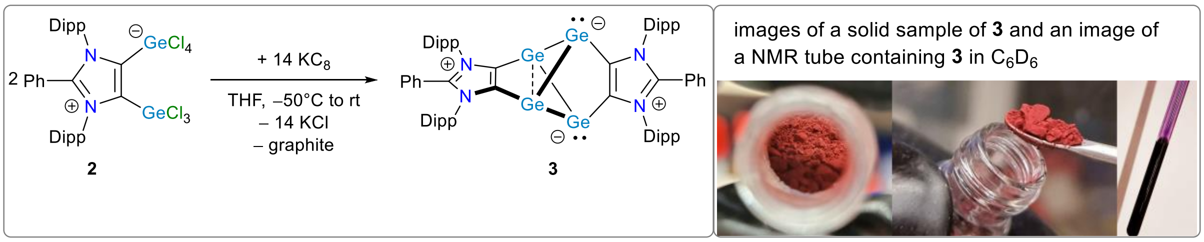

To a Schlenk tube containing **2** (1.50 g, 1.75 mmol) and KC$_8$ (1.70 g, 12.20 mmol) was added 50 mL of THF at –50 °C. The reaction mixture was brought to rt and further stirred for 12h. The resulting dark suspension was filtered. The volatiles from the violet filtrate were removed under vacuum to obtain Ge$_4$-cluster [(ADC)Ge$_2$]$_2$ (**3**) as a Venetian red solid in 97% (1.03 g, 0.85 mmol) yield. Single crystals suitable for sc-XRD were obtained by a slow diffusion of *n*-heptane into a saturated PhF and Me-THF solution of **3**. **MP:** 115 °C (dec.). Elemental analysis (%) calculated for $C_{66}H_{78}N_4Ge_4$ (1217.9): C 65.09, H 6.46, N 4.60; found: C 65.42, H 6.45, N 4.69. **^{1}H NMR** (600 MHz, THF-*d*$_8$, 298 K) 7.39–7.34 (m, 4H, *p*-C$_6$*H*$_3$), 7.23 (d, *J* = 7.8 Hz, 4H, *m*-C$_6$*H*$_3$), 7.20 (d, *J* = 7.8 Hz, 4H, *m*-C$_6$*H*$_3$), 7.14–7.10 (m, 1H, C$_6$*H*$_5$), 7.08–6.99 (m, 7H, C$_6$*H*$_5$), 6.84 (d, *J* = 7.4 Hz, 2H, C$_6$*H*$_5$), 2.84 (sept, *J* = 7.0 Hz, 4H, C*H*(CH$_3$)$_2$), 2.70 (sept, *J* = 6.9 Hz, 4H, C*H*(CH$_3$)$_2$), 1.37 (d, *J* = 6.8 Hz, 12H, CH(C*H*$_3$)$_2$), 1.20 (d, *J* = 6.7 Hz, 12H, CH(C*H*$_3$)$_2$), 1.00 (d, *J* = 6.9 Hz, 12H, CH(C*H*$_3$)$_2$), 0.91 (d, *J* = 6.9 Hz, 12H, CH(C*H*$_3$)$_2$) ppm. **^{13}C{^{1}H} NMR** (151 MHz, THF-*d*$_8$, 298 K) 188.9 (Ge*C*), 152.1, 147.8, 146.7, 146.1, 141.7, 138.1 (N*C*N, *C*$_6$H$_3$, *C*$_6$H$_5$), 134.2 (*m*-*C*$_6$H$_3$), 130.7, 130.6 (*p*-*C*$_6$H$_3$), 130.3 (*p*-*C*$_6$H$_5$), 129.9, 129.7, 129.6, 128.9, 128.8, 126.3, 125.3 (*C*$_6$H$_5$), 125.2, 125.1 (*m*-*C*$_6$H$_3$), 29.8, 29.7 (*C*H(CH$_3$)$_2$), 26.8, 26.4, 23.5, 23.2 (CH(*C*H$_3$)$_2$) ppm. UV-Vis (in THF) λ (nm) (ε (M^{-1} cm^{-1})): 220 (19647), 240 (22531), 272 (28557), 321 nm (25982), 551 (4983).

Synthesis of [(ADC)Ge{Ge(TEMPO)}]$_2$ (4)

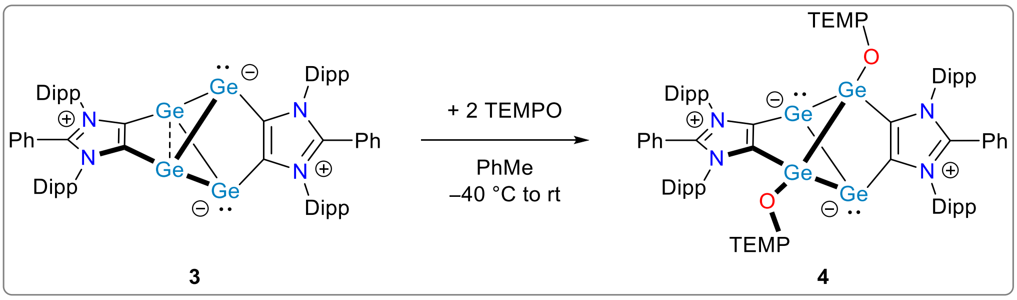

To a Schlenk tube containing **3** (0.45 g, 0.37 mmol) and TEMPO (0.12 g, 0.74 mmol) was added 10 mL of toluene at –40 °C. The dark green solution was brought to rt and stirred for 12h. The volatiles from the solution were removed under vacuum and the obtained green solid was washed with 50 mL *n*-hexane to obtain [(ADC)Ge(Ge{TEMPO})]$_2$ (**4**) a dark green solid in 72% (0.41 g, 0.27 mmol) yield. Single crystals suitable for sc-XRD were obtained by a slow diffusion of *n*-heptane into a saturated toluene solution of **4**. **MP:** 190 °C (dec.). Elemental analysis (%) calculated for $C_{84}H_{114}N_6Ge_4O_2$ (1530.4): C 65.93, H 7.51, N 5.49; found: C 65.56, H 7.55, N 5.43. **^{1}H NMR** (500 MHz, THF-*d*$_8$, 298 K): 7.40 (t, *J* = 7.6 Hz, 2H, C_6H_3), 7.35–7.25 (m, 6H, C_6H_3), 7.19–7.08 (m, 4H, C_6H_3), 7.00 (t, *J* = 7.2 Hz, 2H, *p*-C_6H_5), 6.86 (t, *J* = 7.9 Hz, 4H, *m*-C_6H_5), 6.64 (d, *J* = 8.5 Hz, 4H, *o*-C_6H_5), 3.46 (sept, *J* = 6.4 Hz, 2H, C*H*(CH$_3$)$_2$), 2.68 (sept, *J* = 6.6 Hz, 2H, C*H*(CH$_3$)$_2$), 2.54 (sept, *J* = 6.1 Hz, 2H, C*H*(CH$_3$)$_2$), 2.48 (sept, *J* = 6.1 Hz, 2H, C*H*(CH$_3$)$_2$), 1.50 (d, *J* = 6.9 Hz, 6H, CH(C*H*$_3$)$_2$), 1.47 (d, *J* = 6.7 Hz, 6H, CH(C*H*$_3$)$_2$), 1.44–1.33 (m, 12H, C*H*$_2$), 1.33–1.22 (m, 22H, C*H*$_3$, CH(C*H*$_3$)$_2$), 1.19–1.00 (m, 14H, C*H*$_3$), 0.91 (d, *J* = 6.7 Hz, 6H, CH(C*H*$_3$)$_2$), 0.82 (d, *J* = 7.0 Hz, 6H, CH(C*H*$_3$)$_2$), 0.53 (d, *J* = 7.3 Hz, 6H, CH(C*H*$_3$)$_2$), 0.43 (d, *J* = 6.9 Hz, 6H, CH(C*H*$_3$)$_2$) ppm. **^{13}C{^{1}H} NMR** (126 MHz, THF-*d*$_8$, 298 K): 164.7 (Ge*C*), 154.2, 147.4, 145.0, 144.1, 143.9 (N*C*N, *C*$_6$H$_3$), 136.2, 134.4, 130.4, 130.0, 129.6, 129.3, 128.2, 125.6, 125.4, 125.1, 124.7 (*C*$_6$H$_3$, *C*$_6$H$_5$), 59.5 (N*C*(CH$_3$)$_2$), 41.1 (*C*H$_2$), 32.4, 29.4, 28.8, 27.3 (*C*H(CH$_3$)$_2$), 26.8, 23.8, 23.4, 22.9, 22.4 (CH(*C*H$_3$)$_2$), 18.1, 14.2 ppm. UV-Vis (in THF) λ (nm) (ε (M^{-1} cm^{-1})): 220 (69435), 314 (38120), 410 (9181), 593 (3819).

Synthesis of [(ADC)Ge(Ge{Fe(CO)$_4$})]$_2$ (5)

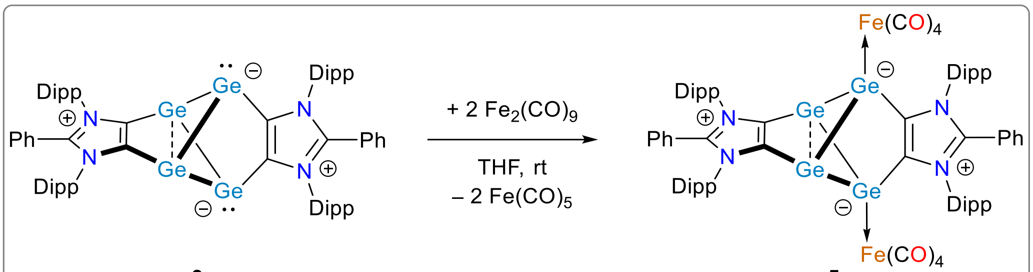

3 **5**

To a Schlenk tube containing **3** (0.30 g, 0.25 mmol) and $Fe_2(CO)_9$ (0.09 g, 0.25 mmol) was added 5 mL of THF and stirred at rt for 12h. The volatiles from the brown solution were removed under vacuum and the brown residue washed with 15 mL *n*-hexane to obtain [(ADC)Ge(Ge{Fe(CO)$_4$})]$_2$ (**5**) a dark brown solid in 65% (0.25 g, 0.16 mmol) yield. Single crystals suitable for sc-XRD were obtained by storing a saturated solution *n*-hexane of **5** at rt for 12h. **MP:** 215 °C (dec.). Elemental analysis (%) calculated for $C_{74}H_{78}N_4Fe_2Ge_4O_8$ (1553.7): C 57.21, H 5.06, N 3.61; found: C 57.55, H 5.03, N 3.56. **^{1}H NMR** (500 MHz, C_6D_6, 298 K): 7.25 (t, *J* = 7.8 Hz, 2H, C_6H_3), 7.10 (d, *J* = 7.8 Hz, 4H, C_6H_3), 7.02–6.99 (m, 2H, C_6H_3), 6.93–6.88 (m, 6H, C_6H_3, C_6H_5), 6.52–6.40 (m, 8H, C_6H_5), 2.92 (sept, *J* = 6.7 Hz, 4H, C*H*(CH$_3$)$_2$), 2.85 (sept, *J* = 6.5 Hz, 4H, C*H*(CH$_3$)$_2$), 1.67 (d, *J* = 6.7 Hz, 6H, CH(C*H*$_3$)$_2$), 1.52 (d, *J* = 6.7 Hz, 6H, CH(C*H*$_3$)$_2$), 0.95 (d, *J* = 6.9 Hz, 6H, CH(C*H*$_3$)$_2$), 0.55 (d, *J* = 6.9 Hz, 6H, CH(C*H*$_3$)$_2$) ppm. **^{13}C{^{1}H} NMR**

(126 MHz, C_6D_6, 298 K): 217.5 (Fe(*C*O)$_4$), 160.4, 146.8, 145.6, 140.8, 131.7 (Ge*C*, *C*$_6$H$_3$, *C*$_6$H$_5$), 131.2, 130.6, 129.7, 129.6, (*C*$_6$H$_5$), 126.1, 125.1 (*C*$_6$H$_3$), 123.4 (N*C*N), 29.1, 29.0, 27.7, 25.1 (*C*H(CH$_3$)$_2$), 23.2, 23.0 (CH(*C*H$_3$)$_2$) ppm. UV-Vis (in THF) λ (nm) (ε (M^{-1} cm^{-1})): 220 (74771), 283 (35191), 357 (12851), 501 (21636), 583 (14030).

Plots of the NMR-Spectra

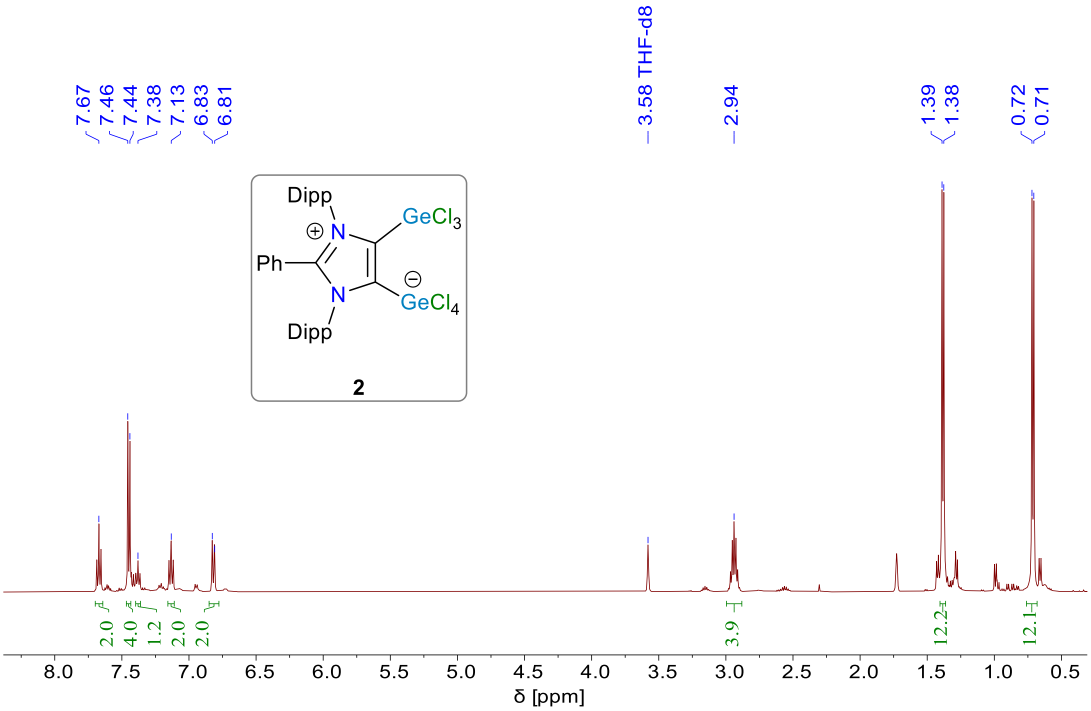

Figure S1. ^{1}H NMR (500 MHz, THF-d_8, 298 K) spectrum of compound **2**.

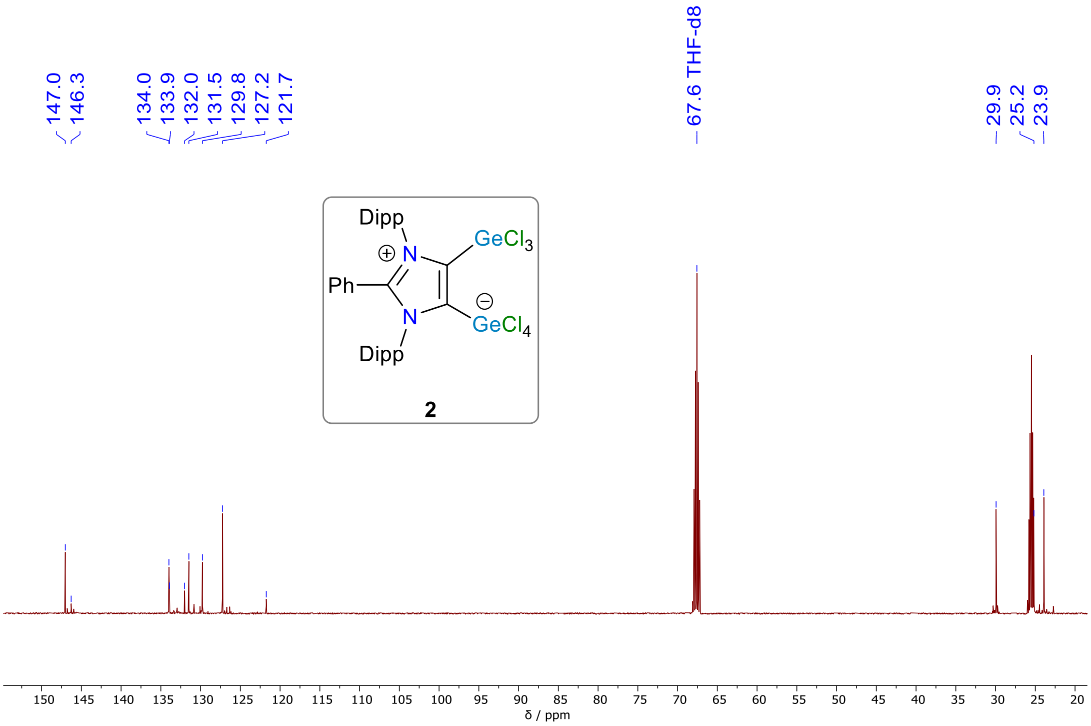

Figure S2. ^{13}C{^{1}H} NMR (126 MHz, THF-d_8, 298 K) of compound **2**.

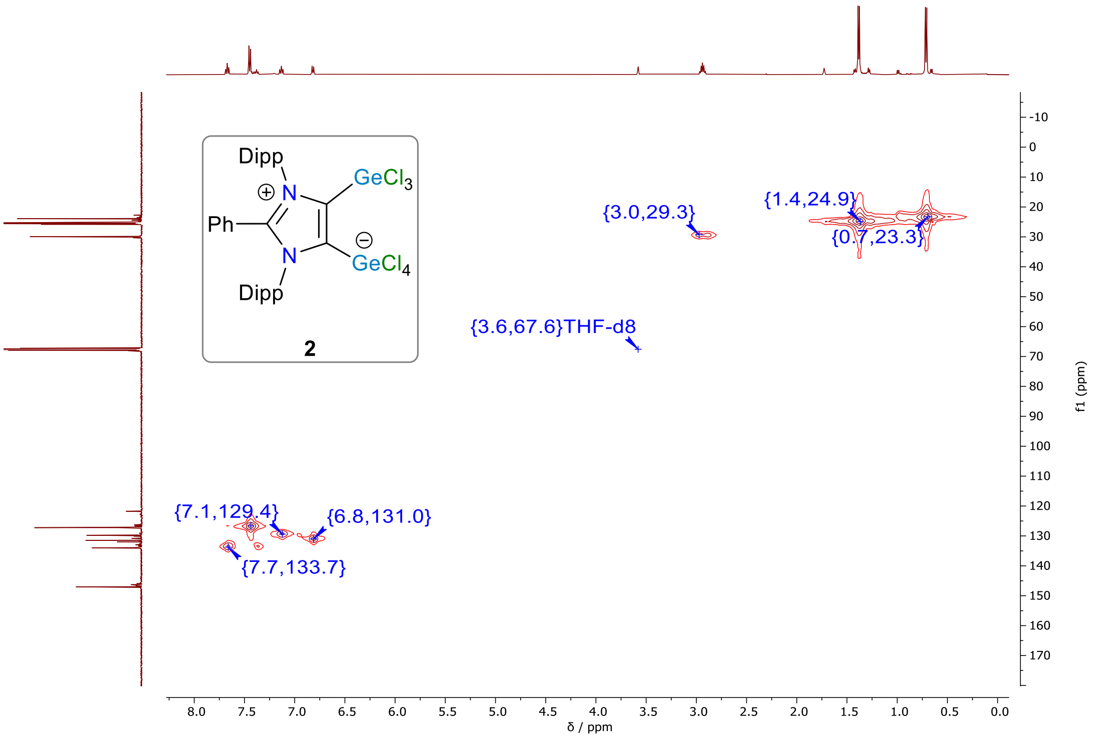

Figure S3. ^{1}H-^{13}C HMQC NMR (THF-d_8, 298 K) spectrum of compound **2**.

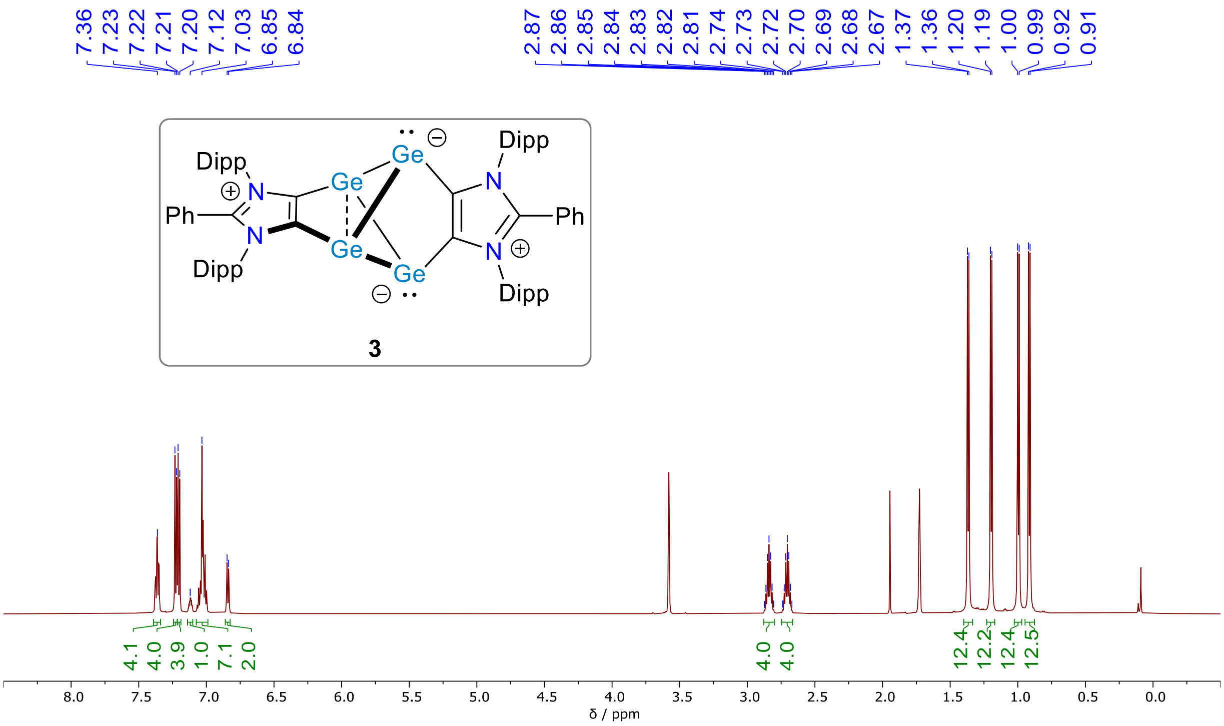

Figure S4. ^{1}H NMR (600 MHz, THF-d_8, 298 K) spectrum of compound **3**.

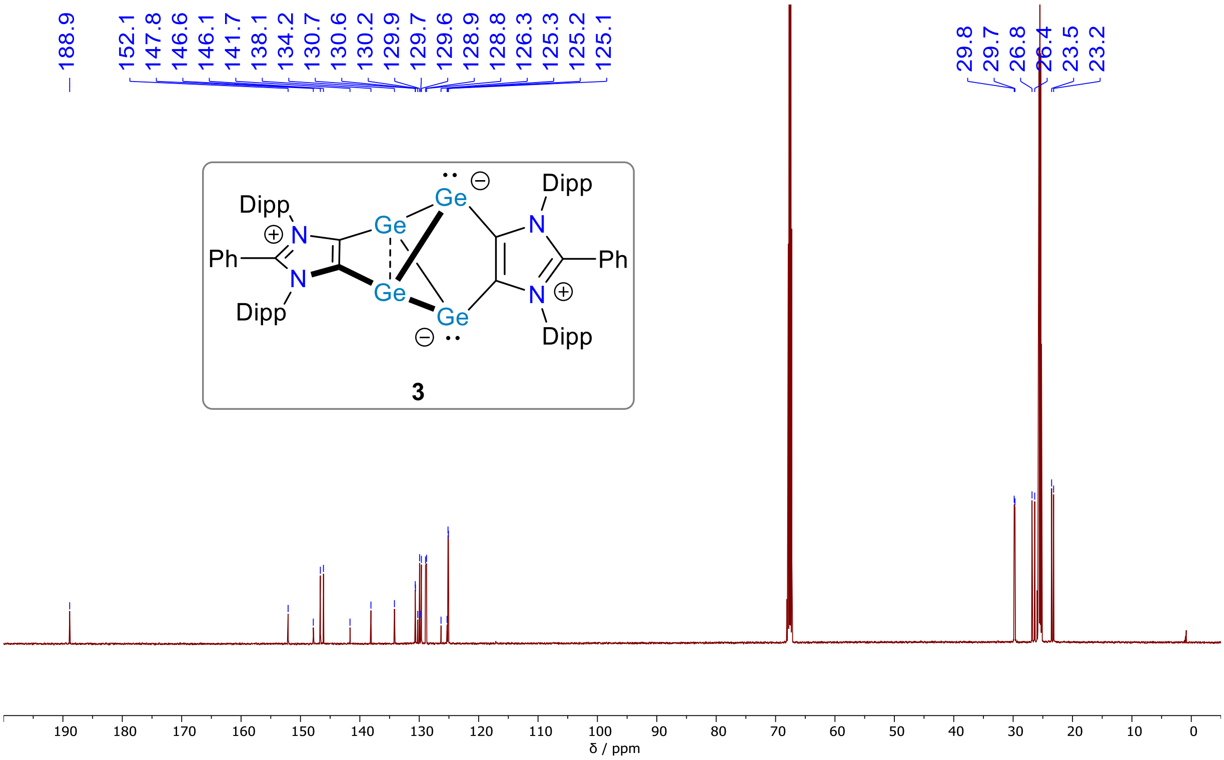

Figure S5. $^{13}C\{^{1}H\}$ NMR (151 MHz, THF-d_8, 298 K) of compound **3** (*Ge-Diradical).

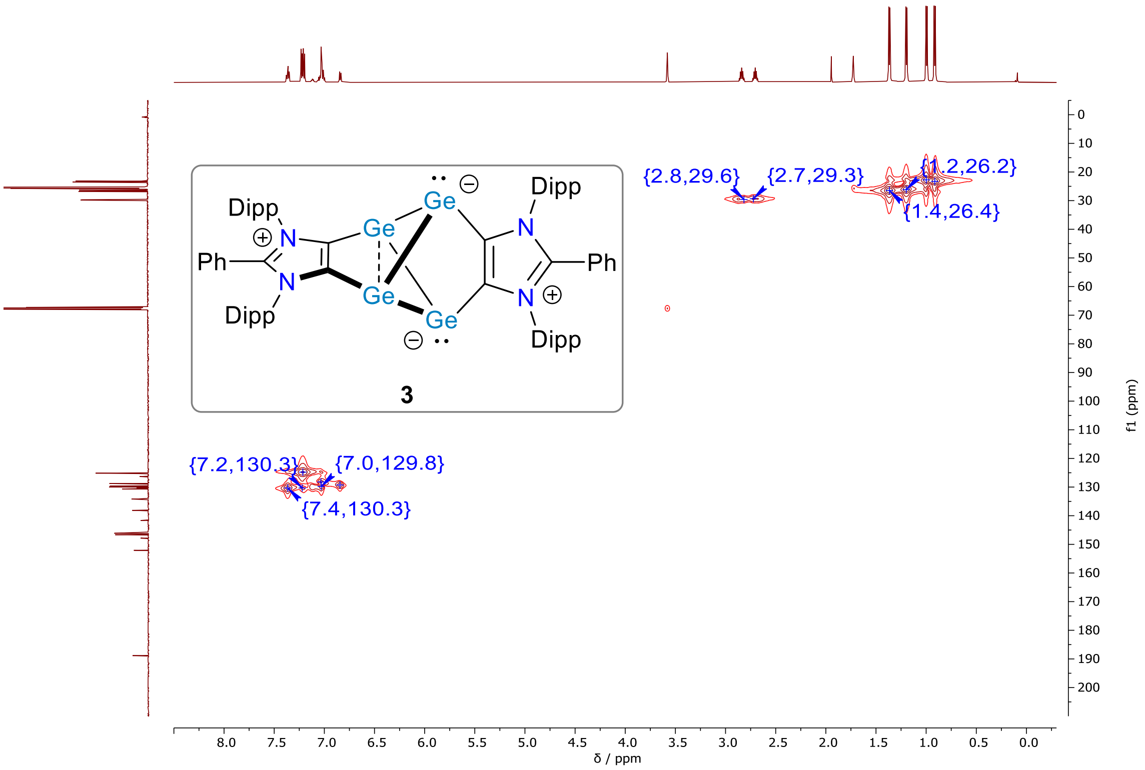

Figure S6. ^{1}H-^{13}C HMQC NMR (THF-d_8, 298 K) spectrum of compound **3**.

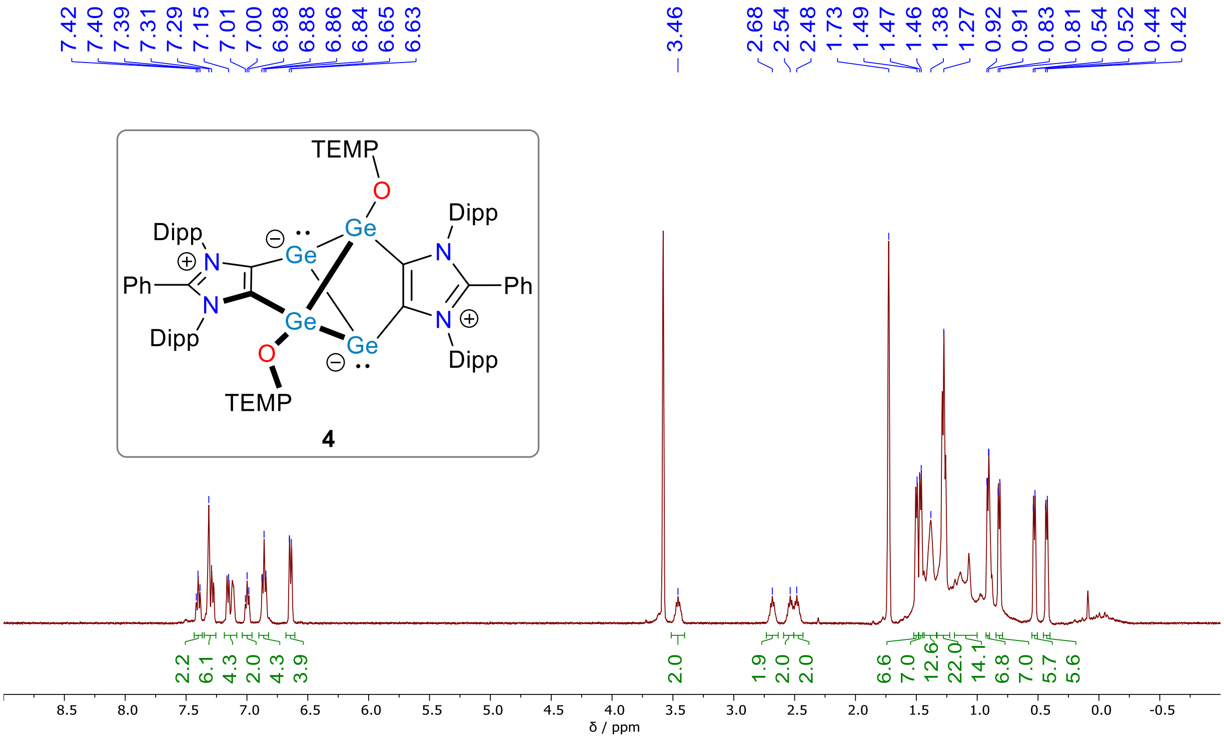

Figure S7. ^{1}H NMR (500 MHz, THF-d_8, 298 K) spectrum of compound **4**.

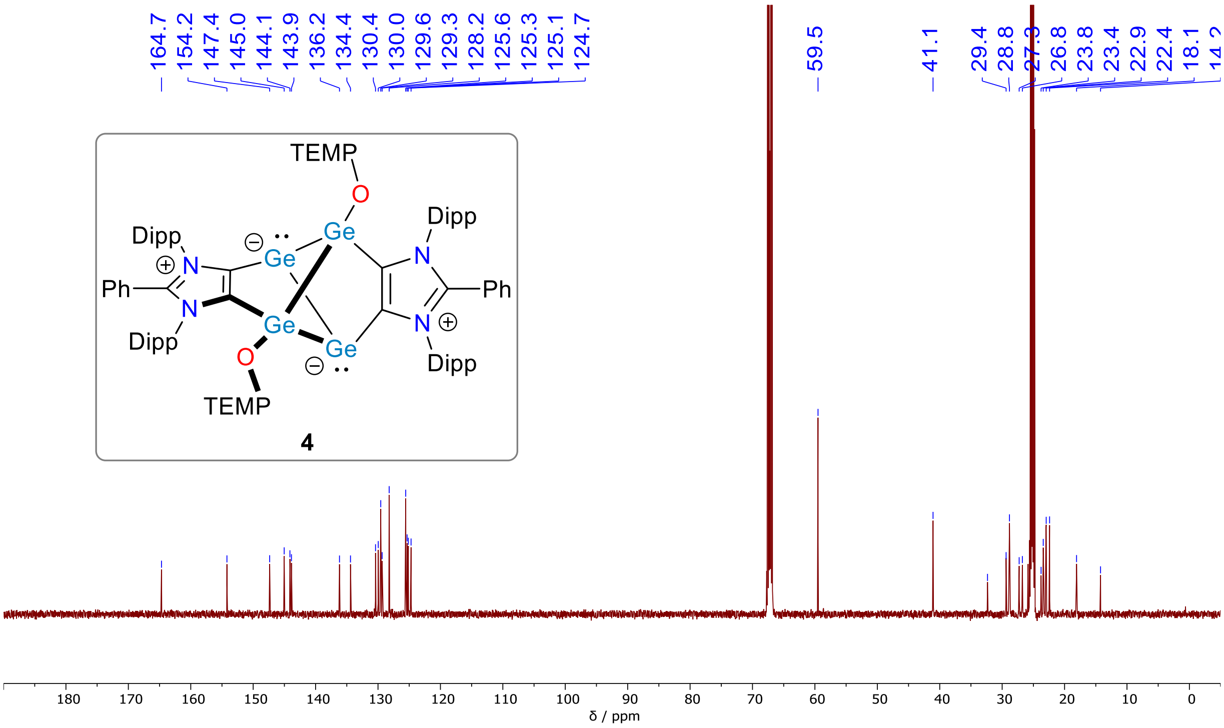

Figure S8. ^{13}C NMR{^{1}H} (126 MHz, THF-d_8, 298 K) of compound **4**.

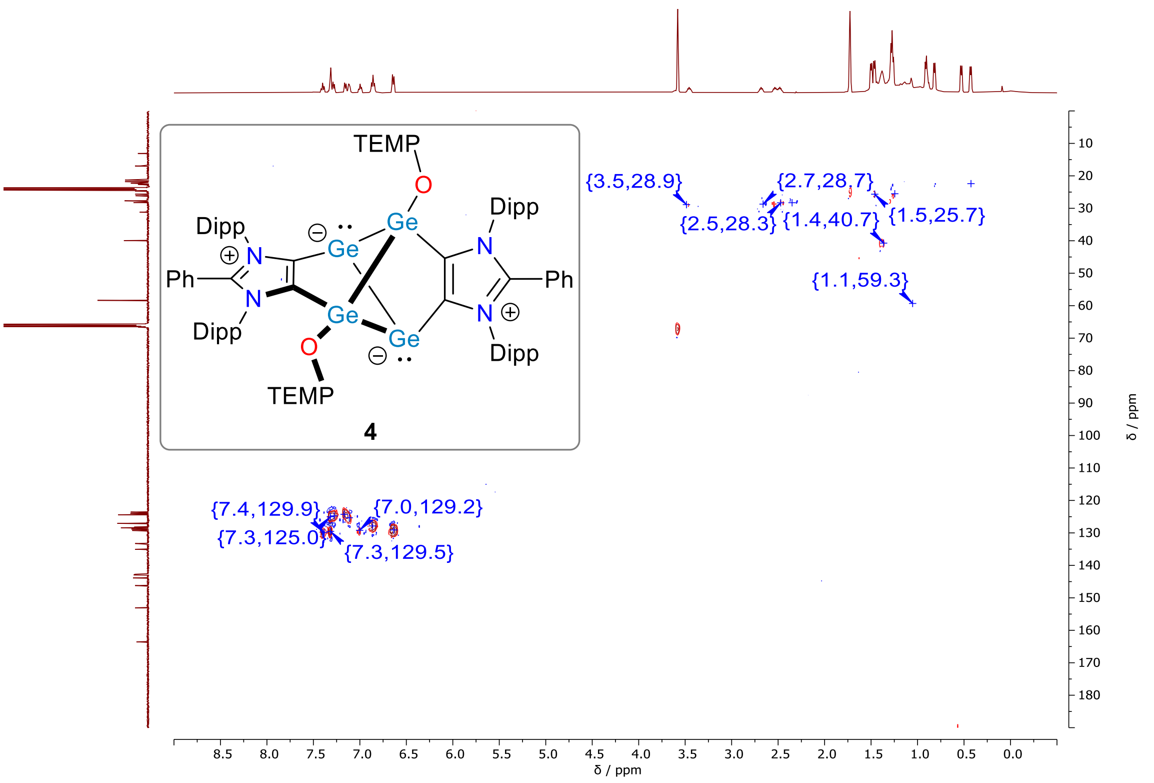

Figure S9. ^{1}H-^{13}C HMQC NMR (THF-d_8, 298 K) spectrum of compound **4**.

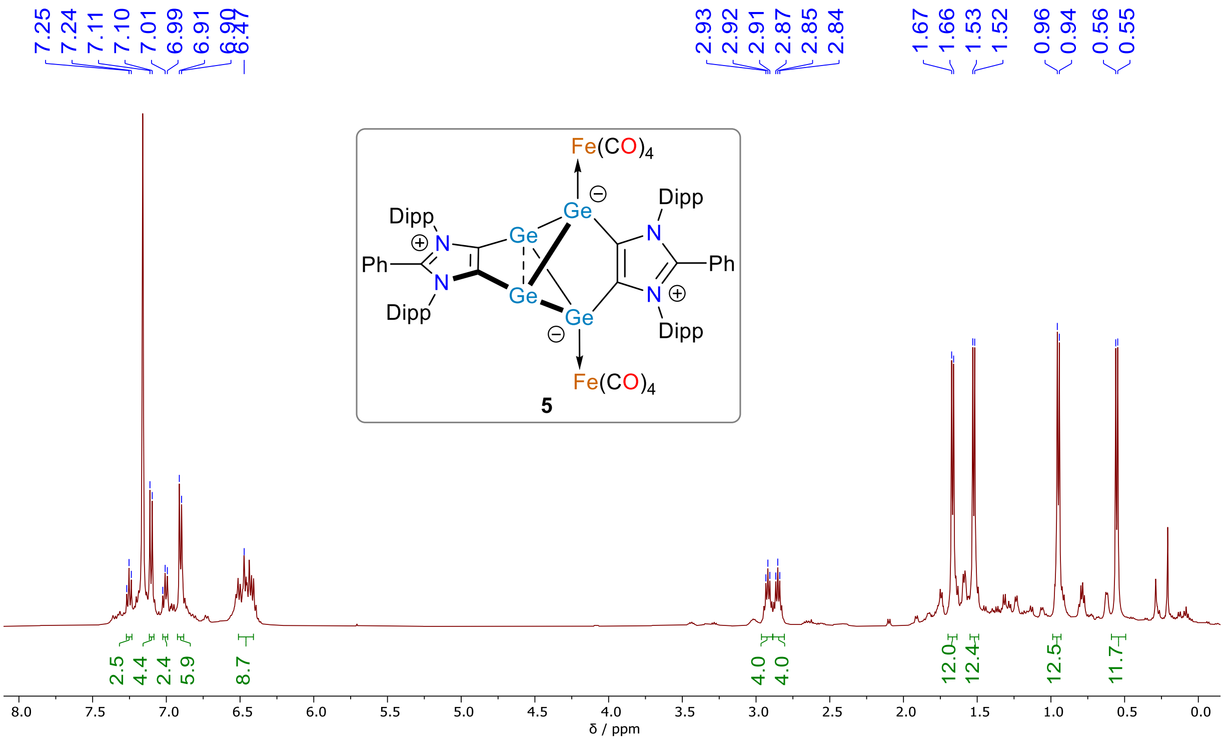

Figure S10. ^{1}H NMR (500 MHz, C_6D_6, 298 K) spectrum of compound **5**.

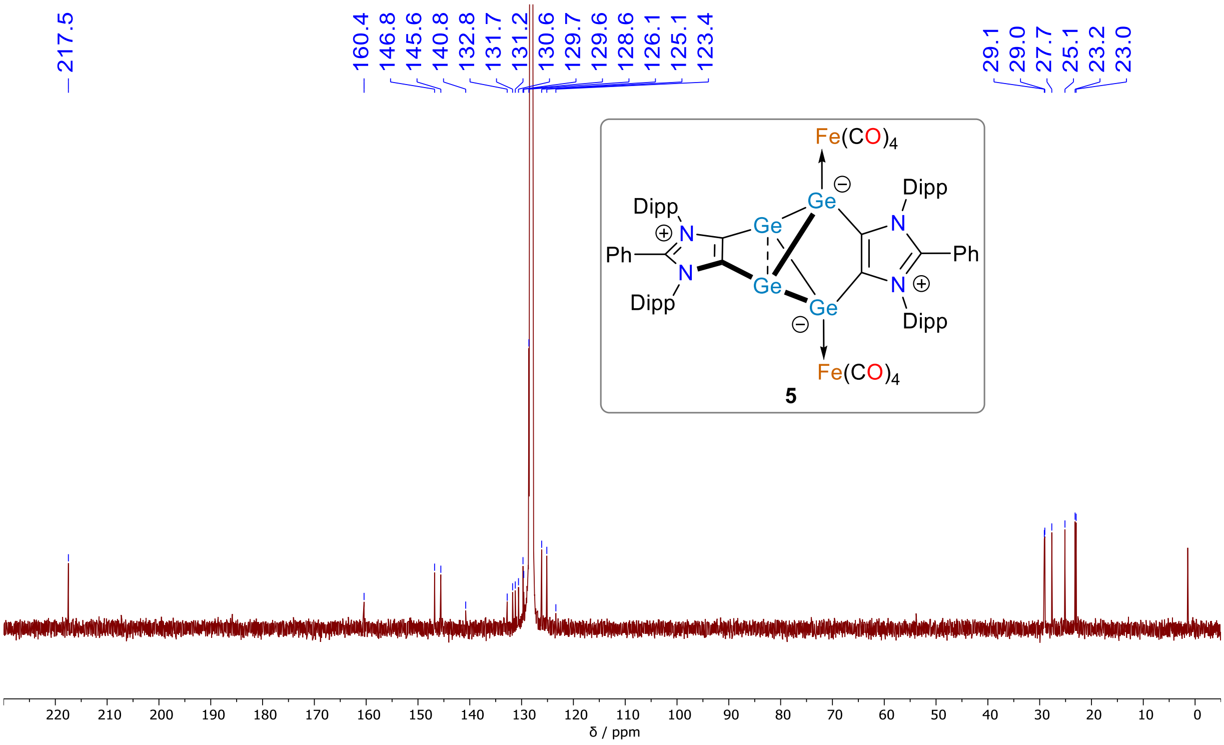

Figure S11. $^{13}C\{^{1}H\}$ NMR (126 MHz, THF-d_8, 298 K) spectrum of compound **5**.

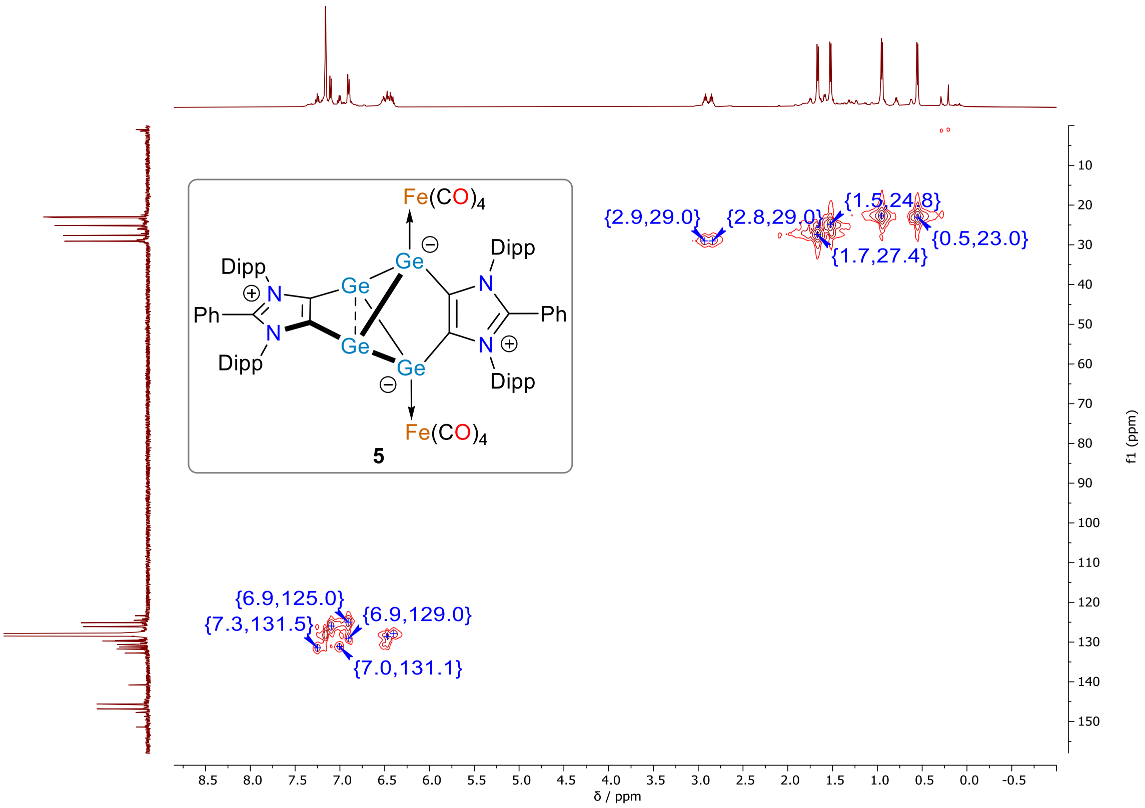

Figure S12. ^{1}H-^{13}C HMQC NMR (C_6D_6, 298 K) spectrum of compound **5**.

Plots UV-Vis Spectra

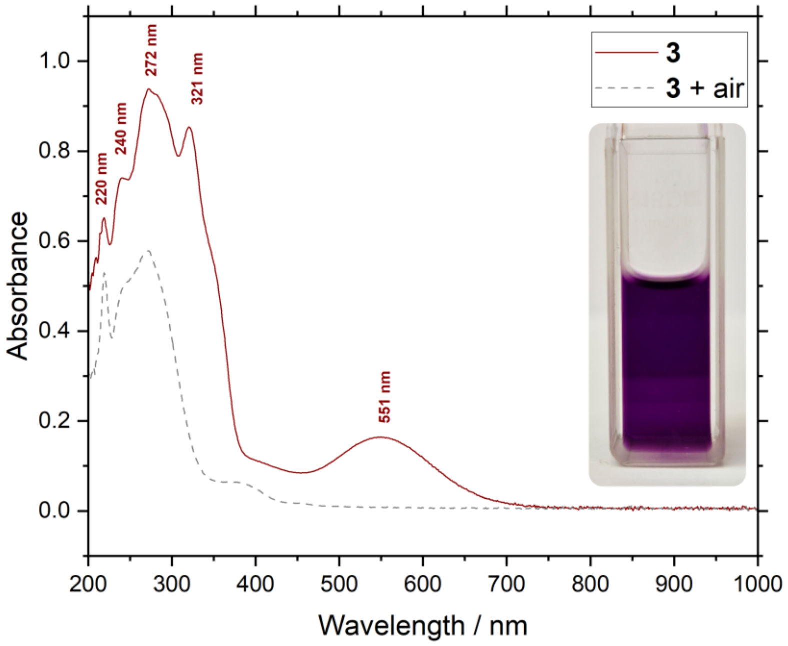

Figure S13. UV- Vis Spectrum of compound **3** (THF, $3{\cdot}10^{-5}$ M, 298 K). Observed absorption bands and calculated molar extinction coefficients ε (L $mol^{-1}cm^{-1}$): [λ_1 = 220 nm (19647); λ_2 = 240 nm (22531); λ_3 = 272 nm (28557); λ_4 = 321 nm (25982); λ_5 = 551 nm (4983)].

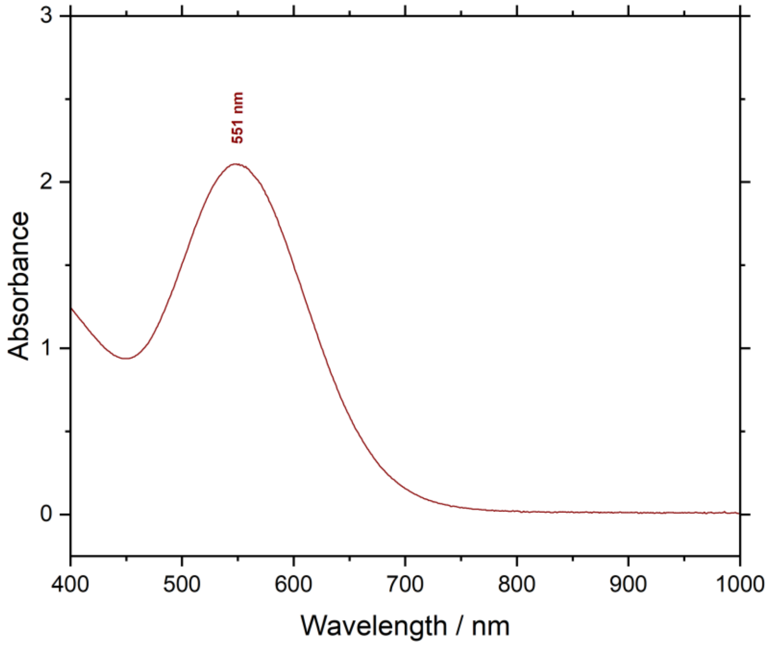

Figure S14. Zoomed UV- Vis Spectrum of compound **3** (THF, $3{\cdot}10^{-4}$ M, 298 K).

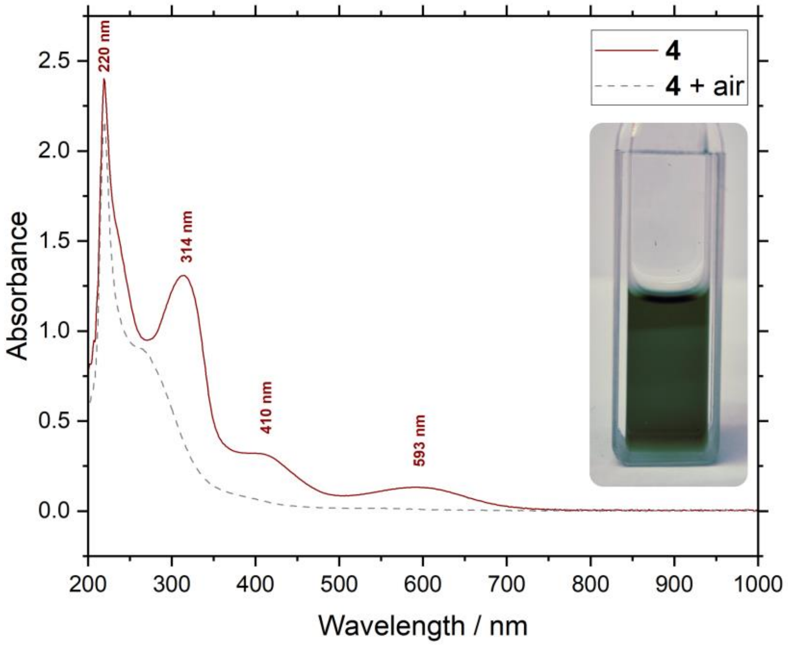

Figure S15. UV- Vis Spectrum of compound **4** (THF, $3 \cdot 10^{-5}$ M, 298 K). Observed absorption bands and calculated molar extinction coefficients ε (L $mol^{-1}cm^{-1}$): [λ_1 = 220 nm (69435); λ_2 = 314 nm (38120); λ_3 = 410 nm (9181); λ_4 = 593 nm (3819)].

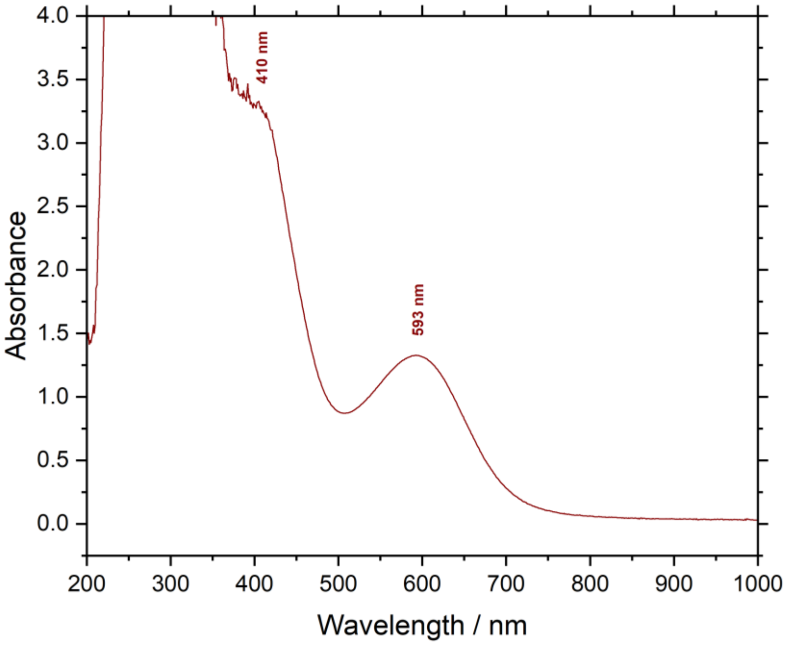

Figure S16. UV- Vis Spectrum of compound **4** (THF, $3 \cdot 10^{-4}$ M, 298 K).

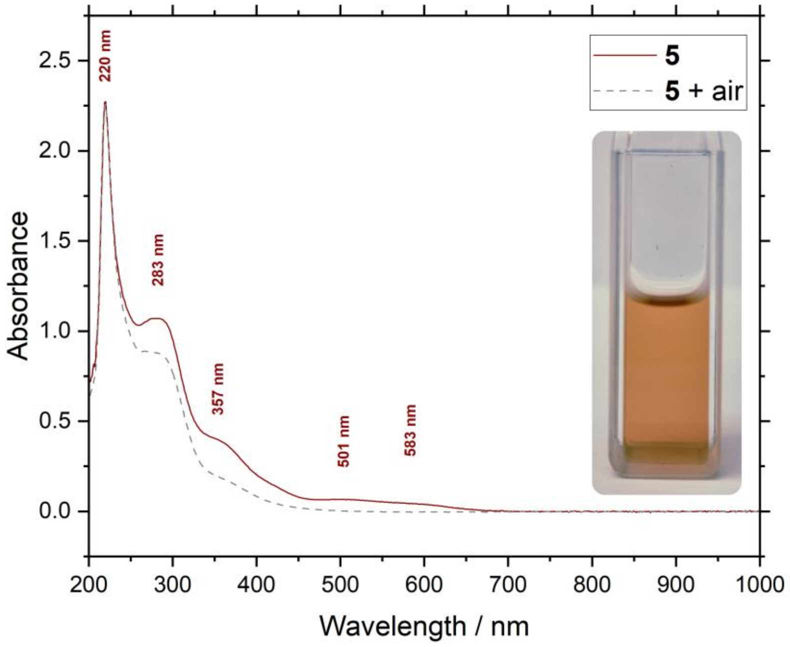

Figure S17. UV- Vis Spectrum of compound **5** (THF, $3{\cdot}10^{-5}$ M, 298 K). Observed absorption bands and calculated molar extinction coefficients ε (L $mol^{-1}cm^{-1}$): [λ_1 = 220 nm (74771); λ_2 = 283 nm (35191); λ_3 = 357 nm (12851); λ_4 = 501 nm (21636); λ_5 = 583 nm (14030)].

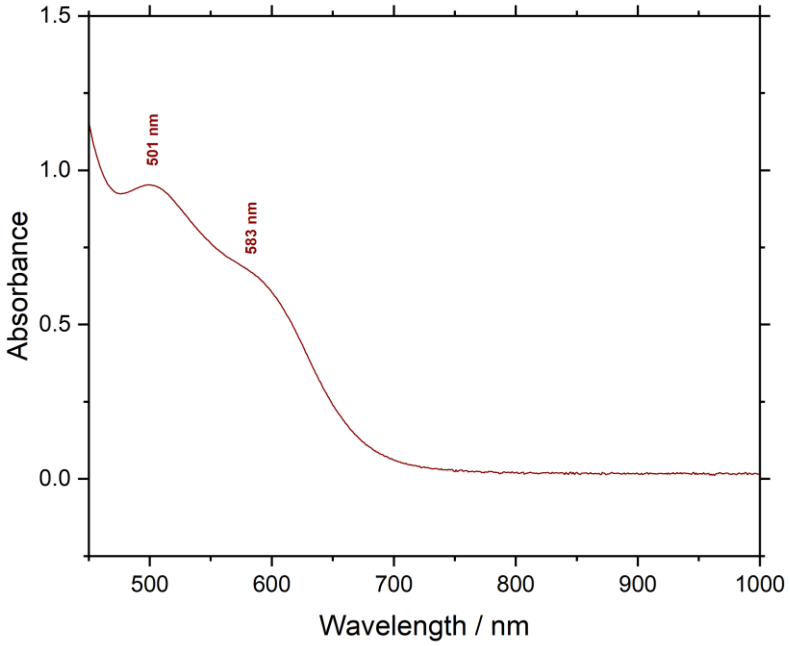

Figure S18. Zoomed UV- Vis Spectrum of compound **5** (THF, $3{\cdot}10^{-4}$ M, 298 K).

Plots of Infrared Spectra

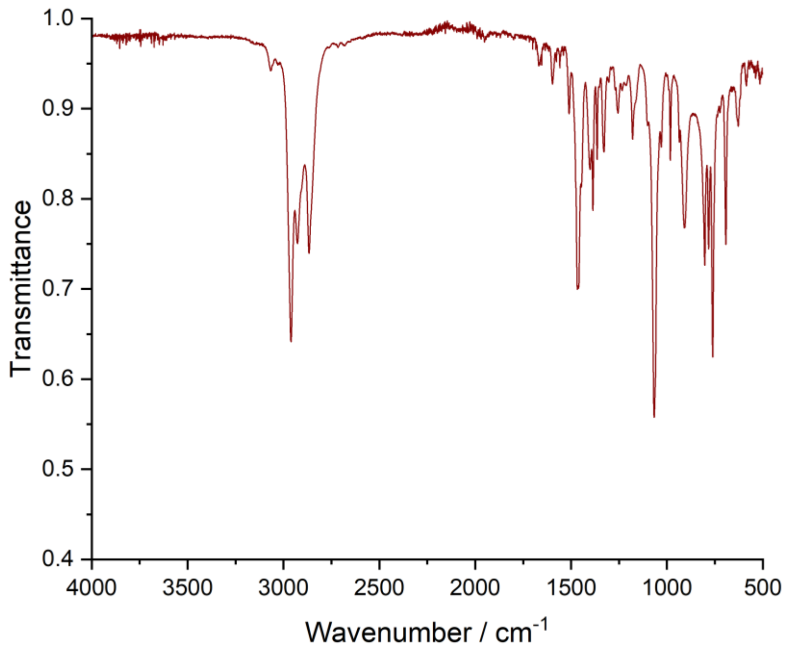

Figure S19. FTIR spectrum of compound **3**.

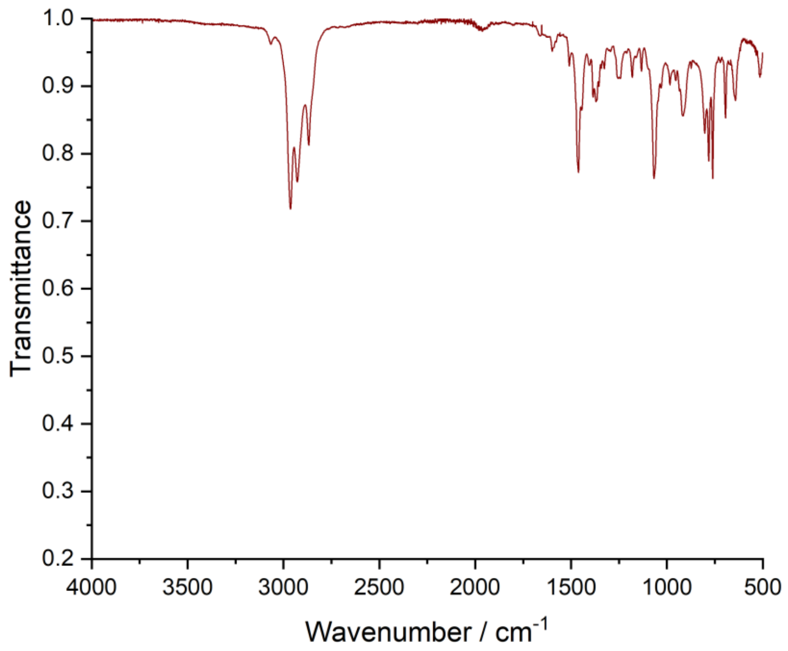

Figure S20. FTIR spectrum of compound **4**.

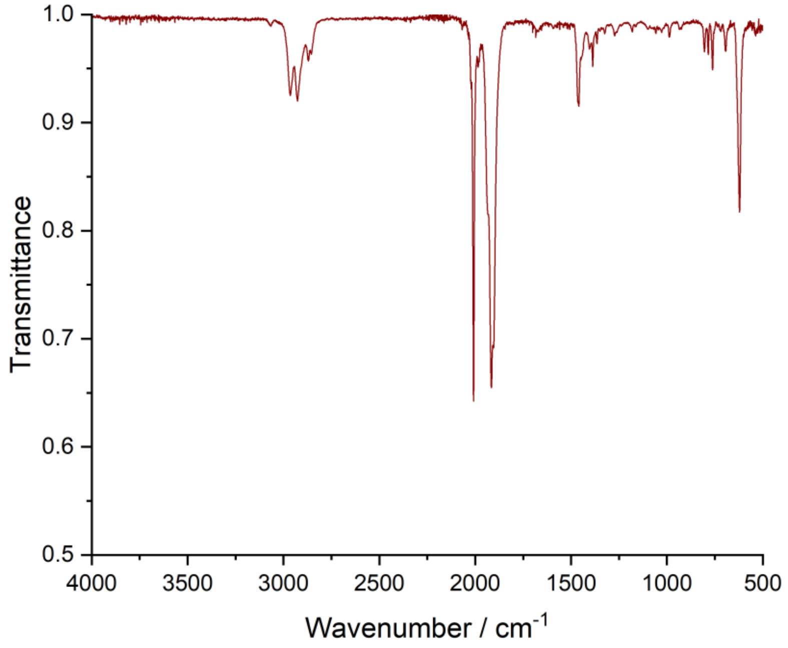

Figure S21. FTIR spectrum of compound **5**.

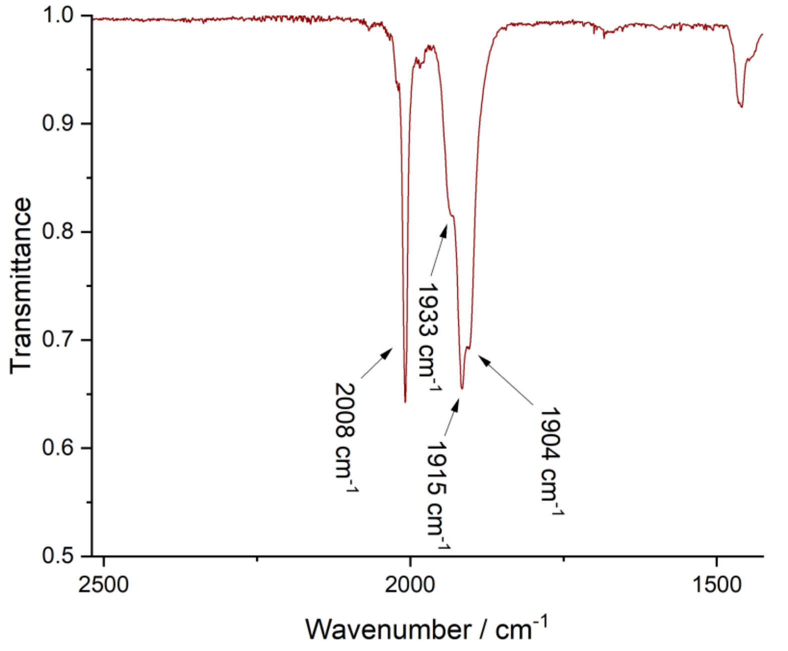

Figure S22. Zoomed FTIR spectrum of compound **5**.

Crystallographic Details

Single crystals were examined on a Rigaku Supernova diffractometer using Cu Kα (λ = 1.54184 Å) or Mo Kα (λ = 0.71073 Å) radiation. The crystals were kept at 100.0(1) K during data collection. Using Olex2,[3] the structures were solved with the ShelXT[4] structure solution program using Intrinsic Phasing and refined with the ShelXL[5] refinement package using Least Squares minimization. **2** was refined as a two component inversion twin. For **3**, a solvent mask was calculated and 350 electrons were found in a volume of 1570 $Å^3$ in one void per unit cell. This is consistent with the presence of two molecules of methyl-THF per formula unit, accounting for 384 electrons per unit cell. Disorder of the germanium atoms (Ge1-4) and one Dipp group (C16/27) over two sites in a ratio of 68 (labelled "A") to 32 (labelled "B"). Disorder of one Dipp group (C4/15), two phenyl groups (C28/33 and C61/66), and partial disorder of one isopropyl group (C44/45) over two sites in a ratio of 61 (labelled "A") to 39 (labelled "B"). For **4**, the solvent molecules were highly disordered. Therefore, a solvent mask was calculated and 214 electrons were found in a volume of 1099 $Å^3$ in one void per unit cell. This is consistent with the presence of two PhMe solvent molecules per asymmetric unit, accounting for 232 electrons per unit cell. Disorder of C26 and C27 over two sites in a ratio of 75:24, strong restraints (SIMU, RIGU) were applied for the disordered atoms. **5** was refined as an inversion twin, with a ratio of 68:32. Disorder of a $Fe(CO)_4$ and a Dipp group with a ratio of 67:33. SIMU and RIGU restraints were used for the disordered atoms.

CCDC 2464588-2464591 contain the supplementary crystallographic data for this paper. These data can be obtained free of charge from The Cambridge Crystallographic Data Centre via www.ccdc.cam.ac.uk/conts/retrieving.html.

Table S1. Crystallographic details of **2** and **3**.

	**2**	**3**
Empirical formula	$C_{40}H_{47}N_2Cl_7Ge_2$	$C_{76}H_{98}Ge_4N_4O_2$
Formula weight	949.12	1389.94
Temperature/K	100.0(1)	100.0(1)
Crystal system	hexagonal	monoclinic
Space group	$P6_5$	$P2_1/n$
a/Å	11.97320(10)	16.9380(3)
b/Å	11.97320(10)	17.6926(2)
c/Å	52.8285(3)	23.8704(4)
β/°	90	91.799(2)
Volume/Å^3	6558.72(12)	7149.89(19)
Z	6	4
ρ_{calc}g/cm^3	1.442	1.291
μ/mm^{-1}	5.858	2.291
F(000)	2904.0	2904.0
Crystal size/mm^3	0.28 × 0.14 × 0.09	0.27 × 0.17 × 0.08
Radiation/Å	Cu Kα (λ = 1.54184)	Cu Kα (λ = 1.54184)
2Θ range for data collection/°	8.528 to 153.49	6.22 to 151.68
Index ranges	$-14 \le h \le 15$, $-13 \le k \le 15$, $-66 \le l \le 66$	$-20 \le h \le 21$, $-22 \le k \le 22$, $-29 \le l \le 30$
Reflections collected	84369	146211
Independent reflections	9159 [R_{int} = 0.0576, R_{sigma} = 0.0225]	14831 [R_{int} = 0.0656, R_{sigma} = 0.0295]
Reflections with $I > 2\sigma(I)$	9122	12687
Data/restraints/parameters	9159/1/470	14831/2099/1053
Goodness-of-fit on F^2	1.051	1.060
Final R indexes [$I > 2\sigma(I)$]	R_1 = 0.0266, wR_2 = 0.0699	R_1 = 0.0570, wR_2 = 0.1346
Final R indexes [all data]	R_1 = 0.0268, wR_2 = 0.0700	R_1 = 0.0650, wR_2 = 0.1389
Largest diff. peak/hole / e Å^{-3}	1.04/-0.31	0.69/-0.46
Flack parameter	0.022(12)	
CCDC number	2464588	2464589

Table S2. Crystallographic details of **4** and **5**.

	**4**	**5**
Empirical formula	$C_{183}H_{243}Ge_8N_{12}O_4$	$C_{74}H_{78}Fe_2Ge_4N_4O_8$
Formula weight	3255.60	1553.46
Temperature/K	100.00(10)	100.00(10)
Crystal system	monoclinic	monoclinic
Space group	$P2_1/n$	P21
a/Å	18.41734(17)	14.1872(4)
b/Å	45.9433(3)	17.5793(5)
c/Å	20.50773(15)	15.2080(5)
β/°	98.0330(8)	106.173(3)
Volume/$Å^3$	17182.4(2)	3642.79(19)
Z	4	2
$\rho_{calc}g/cm^3$	1.259	1.416
μ/mm^{-1}	1.989	5.413
F(000)	6852.0	1588.0
Crystal size/mm^3	0.12 × 0.088 × 0.026	0.18 × 0.07 × 0.03
Radiation/Å	Cu Kα (λ = 1.54184)	Cu Kα (λ = 1.54184)
2Θ range for data collection/°	4.758 to 154.78	6.05 to 152.422
Index ranges	-23 ≤ h ≤ 21, -50 ≤ k ≤ 56, -25 ≤ l ≤ 25	-17 ≤ h ≤ 17, -22 ≤ k ≤ 21, -17 ≤ l ≤ 19
Reflections collected	170966	29832
Independent reflections	34718 [R_{int} = 0.0336, R_{sigma} = 0.0271]	14527 [Rint = 0.0452, Rsigma = 0.0624]
Reflections with $I > 2\sigma(I)$	29561	11893
Data/restraints/parameters	34718/60/1798	14527/918/1033
Goodness-of-fit on F^2	1.014	1.019
Final R indexes [$I > 2\sigma(I)$]	R_1 = 0.0305, wR_2 = 0.0684	R1 = 0.0493, wR2 = 0.1184
Final R indexes [all data]	R_1 = 0.0388, wR_2 = 0.0713	R1 = 0.0652, wR2 = 0.1292
Largest diff. peak/hole / e $Å^{-3}$	0.38/-0.37	1.03/-0.56
Flack parameter		0.318(8)
CCDC number	2464590	2464591

Molecular Structures

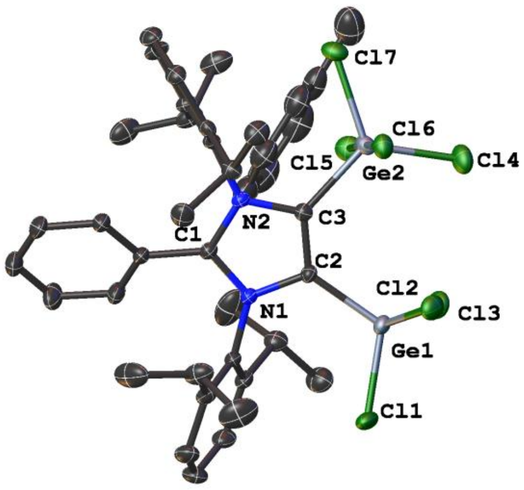

Figure S23. Molecular structure of **2**. H atoms are omitted for clarity. Thermal ellipsoids are shown with 50% probability. Selected bond lengths (Å) and angles (°): C2–C3 1.373(4), Ge1–C2 1.944(3), Ge2–C3 1.995(3), Ge1–Cl1 2.112(1), Ge1–Cl2 2.109(1), Ge1–Cl3 2.117(1), Ge2–Cl4 2.164(1), Ge2–Cl5 2.337(1), Ge2–Cl6 2.269(1), Ge2–Cl7 2.150(1) and C3–C2–Ge1 129.0(2), C2–C3–Ge2 130.7(2), C2–Ge1–Cl1 116.1(1), C3–Ge2–Cl4 124.2(1), C3–Ge2–Cl5 85.0(1), Cl5–Ge2–Cl6 177.3(1).

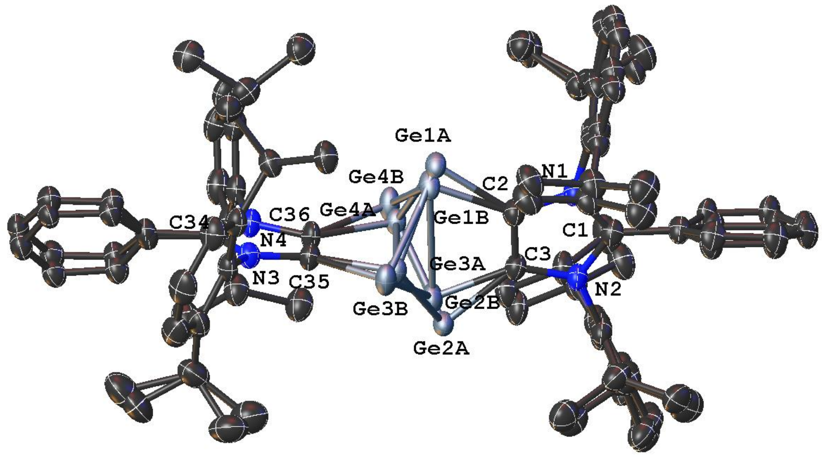

Figure S24. Molecular structure of **3**. H atoms are omitted for clarity. Thermal ellipsoids are shown with 50% probability. Selected bond lengths (Å) and angles (°): C2–C3 1.377(5), C35–C36 1.371(5), Ge1A–C2 2.049(4), Ge2A–C3 2.067(3), Ge3A–C35 2.014(3), Ge4A–C36 1.996(3), Ge1A–Ge3A 2.517(2), Ge1A–Ge4A 2.564(2), Ge2A–Ge3A 2.572(1), Ge2A–Ge4A 2.514(1), Ge3A–Ge4A 2.752(4) and C3–C2–Ge1A 124.8(2), C2–C3–Ge2A 127.6(2), C36–C35–Ge3A 108.5(2), C35–C36–Ge4A 111.6(2), C2–Ge1A–Ge3A 81.7(1), C3–Ge2A–Ge4A 79.8(1), C2–Ge1A–Ge4A 82.3(1), C3–Ge2A–Ge3A 81.7(1), Ge1A–Ge3A–Ge2A 97.5(1), Ge1A–Ge4A–Ge2A 97.8(1), Ge3A–Ge1A–Ge4A 65.6(1), Ge3A–Ge2A–Ge4A 65.5(1), C35–Ge3A–Ge1A 97.3(1), C36–Ge4A–Ge1A 97.9(1), C35–Ge3A–Ge2A 101.9(1), C36–Ge4A–Ge2A 100.5(1).

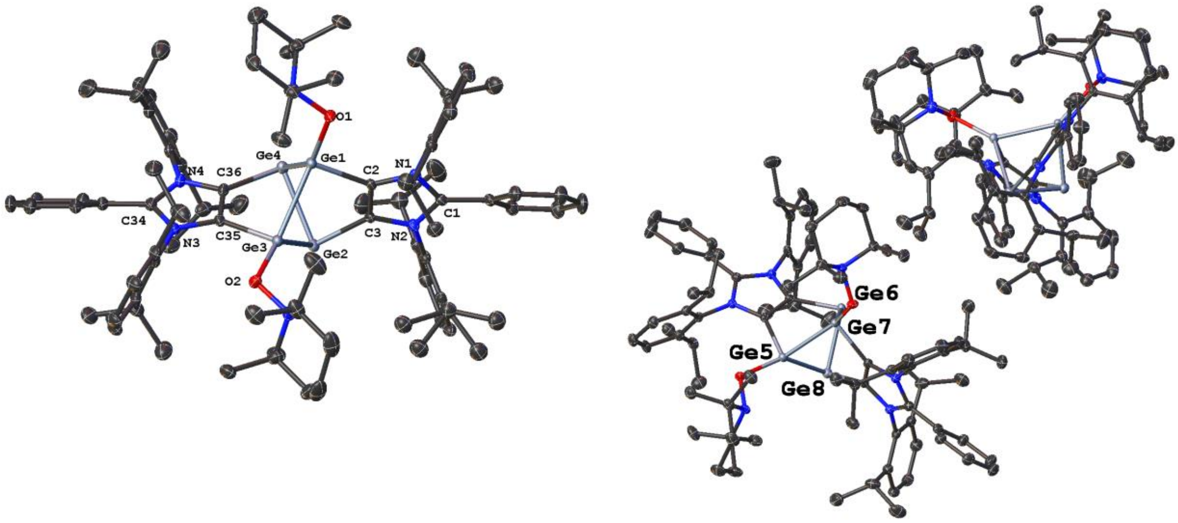

Figure S25. Molecular structure of **4**. H atoms are omitted for clarity. Thermal ellipsoids are shown with 50% probability. Selected bond lengths (Å) and angles (°): C2–C3 1.372(2), Ge1–O1 1.858(1), Ge1–Ge3 2.568(1), Ge1…Ge2 3.337(1), Ge1–Ge4 2.529(1), Ge3…Ge4 3.302(2), Ge1–Ge4–Ge2 82.4(1), Ge1–Ge4–Ge3 50.1(1), Ge1–Ge3–Ge4 49.1(1), Ge4–Ge1–Ge3 80.8(1), Ge3–Ge4–C36 59.7(1), Ge2–Ge4–Ge3–Ge1 119.0(1).

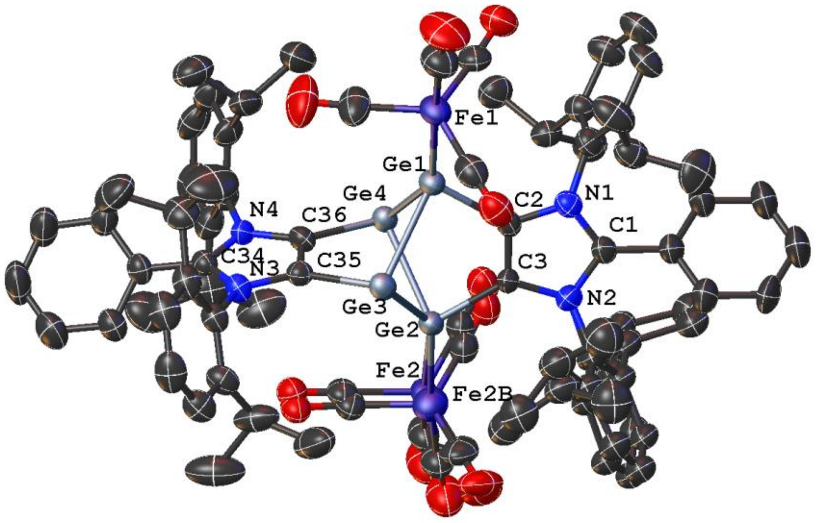

Figure S26. Molecular structure of **5**. H atoms are omitted for clarity. Thermal ellipsoids are shown with 50% probability. Selected bond lengths (Å) and angles (°): C2–C3 1.346(11), Ge1–Fe1 2.360(2), Ge1–Ge3 2.594(1), Ge1…Ge2 3.482(1), Ge1–Ge4 2.462(1), Ge3…Ge4 2.967(1), Ge2–Fe2 2.363(4), Ge1–Ge4–Ge2 87.4(1), Ge1–Ge4–Ge3 56.2(1), Ge1–Ge3–Ge4 52.0(1), Ge4–Ge1–Ge3 71.8(1), Ge3–Ge4–C36 65.7(2), Ge2–Ge4–Ge3–Ge1 117.3(1).

Quantum Chemical Calculations

The molecular structure of $[(ADC)Ge_2]_2$ (**3**) in the free state (modeled as an isolated molecule) was optimized at the PBEh-3c level of theory.[6] All calculations, unless otherwise noted, were performed with the Orca software package versions 6.0.0 and 6.0.1.[7] The solid state structures were used as initial approximations. The target convergence for geometry optimizations was set to *TightOpt* level. In cases of small deviations from higher symmetry the optimized structures were additionally symmetrized. For all Orca calculations, we also used the *TightSCF* and *DefGrid3* settings, as well as the RIJCOSX accelerating approximation.[8] whenever possible. Closed-shell singlet (abbreviated further as **CS**, using the restricted Kohn-Sham formalism RKS), open-shell singlet diradical (**OS**, broken-symmetry unrestricted Kohn-Sham, UKS), and triplet (**T**, UKS) electronic structures were tested using an appropriate method. The results are summarized in Table S3. The optimized molecular structure of **3** for the **CS** singlet solution is shown in Figure S27. Figure S28 shows the optimized molecular structure of **3** in the first triplet (**T**) state.

Fractional occupation weighted density (FOD) calculations[9] at the PBE0/def2-TZVPP (T_{el} = 10000 K) level of theory[10] were performed for **3** (see Table S3 for the resulting N_{FOD} values). The FOD plot of **3** (Figure S29) shows the spatial distributions of the so-called "hot" electrons.

The complete active space self-consistent field (CASSCF) method coupled with the def2-TZVP basis sets was used to study the static electron correlation in **3**. Different starting orbitals were tested, using canonical orbitals, those from FOD calculations, or natural MP2 orbitals. In the end, the path to the most stable results was as follows (arrows indicate orbital transfers): FOD-PBE0/def2-TZVPP → CASSCF(8,7)/def2-SV(P) CASSCF(2,2)/def2-SV(P) → CASSCF(2,2)/def2-TZVP. Starting from the CASSCF(8,7)/def2-SV(P) level, state-specific (SS-CAASCF) and state-averaged (SA-CASSCF) variants of CASSCF calculations were performed independently. SS-CASSCF calculations were performed to evaluate the diradical character β of **3** (see Table S3) using the literature method.[11] SA-CASSCF calculations were performed to calculate the energy difference between the ground and excited electronic states. Based on the obtained SA-CASSCF wavefunctions, the DLPNO-accelerated FIC-NEVPT2[12] method was used to account for the dynamic electron correlation. The results are summarized in Tables S3–S7.

The TD-DFT calculations were performed at the PBE0/def2-TZVPP level of theory using the TDA approximation and CPCM solution models for the solvents, which have been used in the actual UV-Vis measurements of **3**. The number of calculated roots was limited to 40. Selected transitions and frontier molecular orbitals from these calculations are listed in Tables S8 and S9. The simulated UV-Vis spectrum is shown in Figure S30.

The Natural bond orbital (NBO) analyses[13] were performed using the NBO 7.0.10 program.[14] The required wavefunctions were taken from single-point PBE0/def2-TZVPP calculations using the

optimized most stable structures. The natural charges and Wiberg bond indices for selected atoms and atom pairs of compound **3** are listed in Table S10.

The same wavefunction of **3** was analyzed in terms of the quantum theory of atoms-in-molecules (QTAIM)[15] using AIMAll 19.10.12 software.[16] The calculated molecular graph and plots of the electron density and the Laplacian of the electron density are shown in Figures S31–S36. The results of the QTAIM analysis are given in Table S11.

Additionally, the molecular structure of $[(ADC)Ge(Ge\{Fe(CO)_4\})]_2$ (**5**) was investigated using similar methods. The Equilibrium structure (see Figures S37) was optimized at the RKS-PBEh-3c level of theory. The electron wavefunction was calculated at the PBE0/def2-TZVPP level. Using this wavefunction, NBO (Table S13) and QTAIM analysis (Table S14 and Figures S38–S41) were performed for **5**.

Table S3. Results of the quantum-chemical calculations.[a]

Mol.	E_{DFT}	$\langle S^2 \rangle_{DFT}$	N_{FOD}	y (%)	ΔE_{NEVPT2}	β (%)
**3**	0 (CS), 13.9 (T)	2.028 (D)	2.88	n.a.	42.3 (S→T)	9

[a]E_{DFT} are relative adiabatic energies in kcal/mol obtained from DFT calculations for different electronic solutions (CS closed-shell singlet, OS broken-symmetry open-shell singlet, D doublet, T triplet) indicated in parentheses; $\langle S^2 \rangle_{DFT}$ are expectation values of the spin-squared operator for the respective electronic solutions; N_{FOD} indicates the number of "hot" electrons obtained as a result of the FOD calculation; y is the diradical character (in %) obtained from the UHF occupation numbers; ΔE_{NEVPT2} is the vertical energy difference between the ground and first excited electronic states as calculated by NEVPT2 method; β is diradical character (in %) obtained from the SS-CASSCF calculation; n.a. stands for "not applicable", indicating that the corresponding electronic solution has not been found or is not possible.

Table S4. Electronic structure parameters in the SS-CASSCF calculations.

	CAS Solution
**3**<br>(2,2)<br>def2-TZVP	CAS-SCF STATES FOR BLOCK 0 MULT= 1 NROOTS= 1<br>---<br><br>ROOT 0: E= -11064.8860169060 Eh<br>0.95748 [0]: 20<br>0.04252 [2]: 02<br><br>DENSITY MATRIX<br>--------------<br><br>0 1<br>0 1.914962 -0.000000<br>1 -0.000000 0.085038<br>Trace of the electron density: 2.000000

Table S5. Active space orbitals (isosurfaces 0.05 a.u.) in the SS-CASSCF(2,2) calculation of **3**.

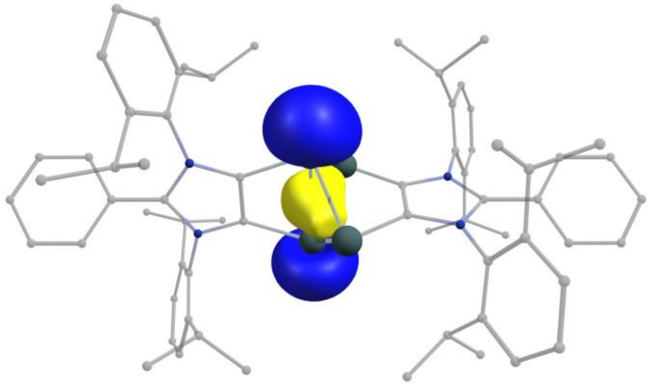<br>HOMO	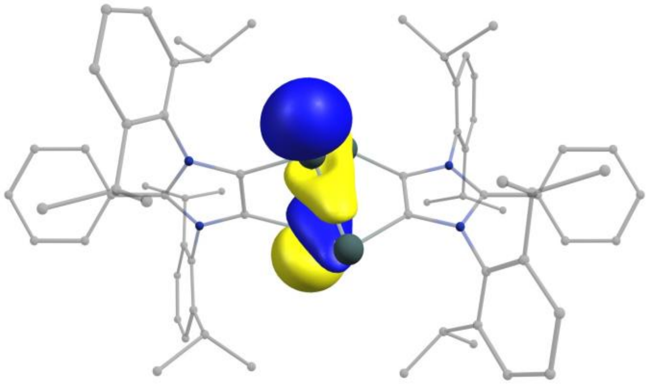<br>LUMO

Table S6. Electronic structure parameters in the SA-CASSCF and NEVPT2 calculations.

	CAS Solution
**3**<br>(2,2)<br>def2-TZVP	CAS-SCF STATES FOR BLOCK 0 MULT= 3 NROOTS= 1<br>---<br><br>ROOT 0: E= -11064.7931444920 Eh<br>1.00000 [0]: 11<br><br>CAS-SCF STATES FOR BLOCK 1 MULT= 1 NROOTS= 1<br>---<br><br>ROOT 0: E= -11064.8688654260 Eh<br>0.99898 [0]: 20<br><br>SA-CASSCF TRANSITION ENERGIES<br>------------------------------<br><br>LOWEST ROOT (ROOT 0 ,MULT 1) = -11064.868865426 Eh -301090.389 eV<br><br>STATE ROOT MULT DE/a.u. DE/eV DE/cm**-1<br>1: 0 3 0.075721 2.060 16618.8<br><br>DENSITY MATRIX<br>--------------<br><br>0 1<br>0 1.498979 0.000000<br>1 0.000000 0.501021<br>Trace of the electron density: 2.000000<br><br>NEVPT2 TRANSITION ENERGIES<br>------------------------------<br><br>LOWEST ROOT (ROOT 0, MULT 1) = -11077.410202858 Eh -301431.656 eV<br><br>STATE ROOT MULT DE/a.u. DE/eV DE/cm**-1<br>1: 0 3 0.067395 1.834 14791.5<br><br>NEVPT2 CORRECTION TO THE TRANSITION ENERGY<br>---<br>STATE ROOT MULT DE/a.u. DE/eV DE/cm**-1<br>1: 0 3 -0.008323 -0.226 -1826.7

Table S7. Active space orbitals (isosurfaces 0.05 a.u.) in the SA-CASSCF(2,2) calculation of **3**.

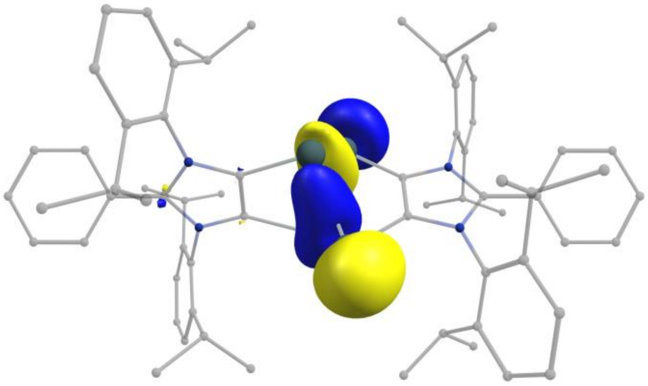 HOMO	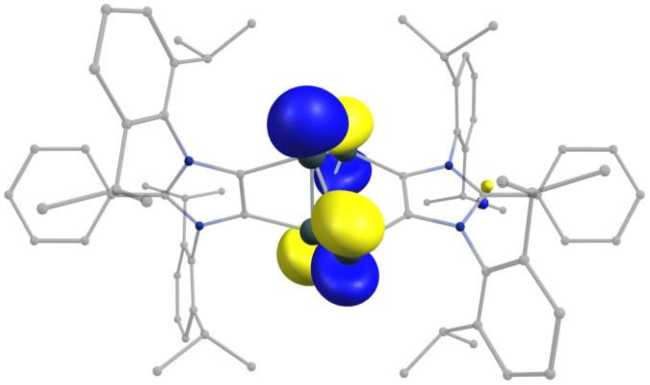 LUMO

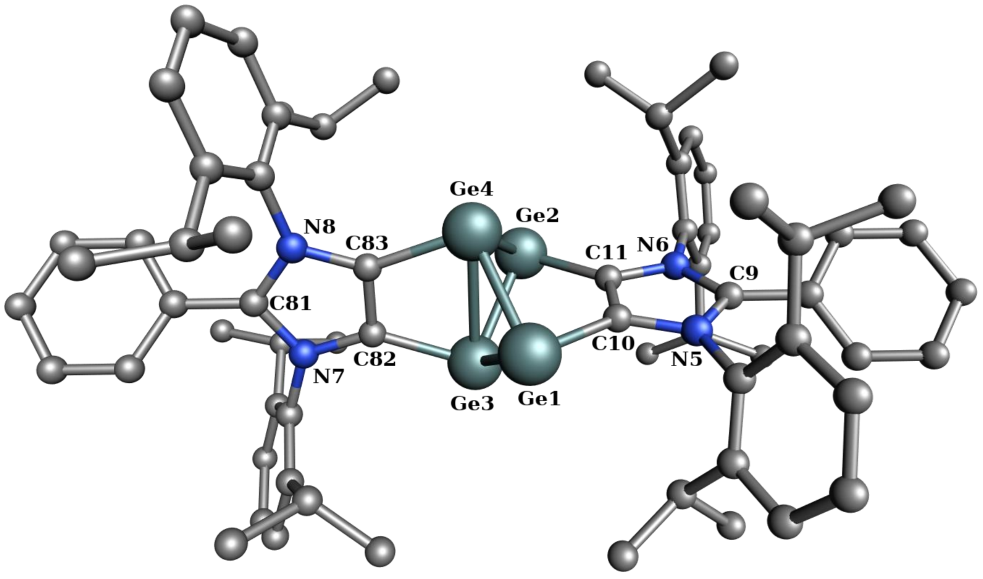

Figure S27. The optimized molecular structure of free **3** (C_2 symmetry) for the **CS** singlet state. The hydrogen atoms are omitted for clarity. The internal working numeration of selected atoms is shown. Selected equilibrium parameters (Å, degrees) are: r(Ge3–Ge1) = 2.527, r(Ge3–Ge2) = 2.546, r(Ge4–Ge1) = 2.546, r(Ge4–Ge2) = 2.527, r(Ge4–Ge3) = 2.747, r(C9–N5) = 1.341, r(C9–N6) = 1.341, r(C10–Ge1) = 2.024, r(C10–N5) = 1.383, r(C11–Ge2) = 2.024, r(C11–N6) = 1.383, r(C11–C10) = 1.386, r(C81–N7) = 1.348, r(C81–N8) = 1.348, r(C82–Ge3) = 2.022, r(C82–N7) = 1.372, r(C83–Ge4) = 2.022, r(C83–N8) = 1.372, r(C83–C82) = 1.361, a(Ge1–Ge3–Ge2) = 98.1, a(Ge3–Ge1–Ge4) = 65.6, a(Ge1–Ge3–Ge4) = 57.6, a(Ge3–Ge1–C10) = 80.1, a(Ge1–Ge3–C82) = 98.7, a(Ge3–Ge2–Ge4) = 65.6, a(Ge2–Ge3–Ge4) = 56.9, a(Ge3–Ge2–C11) = 81.1, a(Ge2–Ge3–C82) = 99.8, a(Ge1–Ge4–Ge2) = 98.1, a(Ge1–Ge4–Ge3) = 56.9, a(Ge4–Ge1–C10) = 81.1, a(Ge1–Ge4–C83) = 99.8, a(Ge2–Ge4–Ge3) = 57.6, a(Ge4–Ge2–C11) = 80.1, a(Ge2–Ge4–C83) = 98.7, a(Ge4–Ge3–C82) = 69.9, a(Ge3–Ge4–C83) = 69.9, a(N5–C9–N6) = 106.1, a(C9–N5–C10) = 111.0, a(C9–N6–C11) = 111.0, a(Ge1–C10–N5) = 127.0, a(Ge1–C10–C11) = 127.0, a(N5–C10–C11) = 105.9, a(Ge2–C11–N6) = 127.0, a(Ge2–C11–C10) = 127.0, a(N6–C11–C10) = 105.9, a(N7–C81–N8) = 106.3, a(C81–N7–C82) = 110.0, a(C81–N8–C83) = 110.0, a(Ge3–C82–N7) = 143.1, a(Ge3–C82–C83) = 110.0, a(N7–C82–C83) = 106.9, a(Ge4–C83–N8) = 143.1, a(Ge4–C83–C82) = 110.0, a(N8–C83–C82) = 106.9, t(Ge4–Ge1–Ge3–Ge2) = 41.9, t(Ge1–Ge3–Ge2–Ge4) = –42.2, t(C10–Ge1–Ge3–Ge2) = –42.8, t(Ge1–Ge3–Ge2–C11) = 40.9, t(Ge3–Ge1–Ge4–Ge2) = –42.2, t(C82–Ge3–Ge1–Ge4) = –59.4, t(Ge3–Ge1–Ge4–C83) = 58.1, t(Ge1–Ge3–Ge4–Ge2) = 127.9, t(C10–Ge1–Ge3–Ge4) = –84.7, t(Ge1–Ge3–Ge4–C83) = –117.0, t(Ge3–Ge1–C10–N5) = –144.7, t(Ge3–Ge1–C10–C11) = 38.4, t(C82–Ge3–Ge1–C10) = –144.1, t(Ge1–Ge3–C82–N7) = –128.2, t(Ge1–Ge3–C82–C83) = 53.6, t(Ge3–Ge2–Ge4–Ge1) = 41.9, t(C82–Ge3–Ge2–Ge4) = 58.1, t(Ge3–Ge2–Ge4–C83) = –59.4, t(Ge2–Ge3–Ge4–Ge1) = –127.9, t(C11–Ge2–Ge3–Ge4) = 83.1, t(Ge2–Ge3–Ge4–C83) = 115.0, t(Ge3–Ge2–C11–N6) = 148.7, t(Ge3–Ge2–C11–C10) = –28.1, t(C82–Ge3–Ge2–C11) = 141.2, t(Ge2–Ge3–C82–N7) = 132.0, t(Ge2–Ge3–C82–C83) = –46.3, t(C10–Ge1–Ge4–Ge2) = 40.9, t(Ge1–Ge4–Ge2–C11) = –42.8, t(C10–Ge1–Ge4–Ge3) = 83.1, t(Ge1–Ge4–Ge3–C82) = 115.0, t(Ge4–Ge1–C10–N5) = 148.7, t(Ge4–Ge1–C10–C11) = –28.1, t(C83–Ge4–Ge1–C10) = 141.2, t(Ge1–Ge4–C83–N8) = 132.0, t(Ge1–Ge4–C83–C82) = –46.3, t(C11–Ge2–Ge4–Ge3) = –84.7, t(Ge2–Ge4–Ge3–C82) = –117.0, t(Ge4–Ge2–C11–N6) = –144.7, t(Ge4–Ge2–C11–C10) = 38.4, t(C83–Ge4–Ge2–C11) = –144.1, t(Ge2–Ge4–C83–N8) = –128.2, t(Ge2–Ge4–C83–C82) = 53.6, t(Ge4–Ge3–C82–N7) = –178.8, t(C83–Ge4–Ge3–C82) = –2.0, t(Ge4–Ge3–C82–C83) = 2.9, t(Ge3–Ge4–C83–N8) = –178.8, t(Ge3–Ge4–C83–C82) = 2.9, t(C10–N5–C9–N6) = –1.0, t(N5–C9–N6–C11) = –1.0, t(C9–N5–C10–Ge1) = –174.9, t(C9–N5–C10–C11) = 2.5, t(C9–N6–C11–Ge2) = –174.9, t(C9–N6–C11–C10) = 2.5, t(Ge1–C10–C11–Ge2) = –8.1, t(Ge1–C10–C11–N6) = 174.5, t(N5–C10–C11–Ge2) = 174.5, t(N5–C10–C11–N6) = –2.9, t(C82–N7–C81–N8) = –0.6, t(N7–C81–N8–C83) = –0.6, t(C81–N7–C82–Ge3) = –176.8, t(C81–N7–C82–C83) = 1.5, t(C81–N8–C83–Ge4) = –176.8, t(C81–N8–C83–C82) = 1.5, t(Ge3–C82–C83–Ge4) = –4.0, t(Ge3–C82–C83–N8) = 177.1, t(N7–C82–C83–Ge4) = 177.1, t(N7–C82–C83–N8) = –1.8.

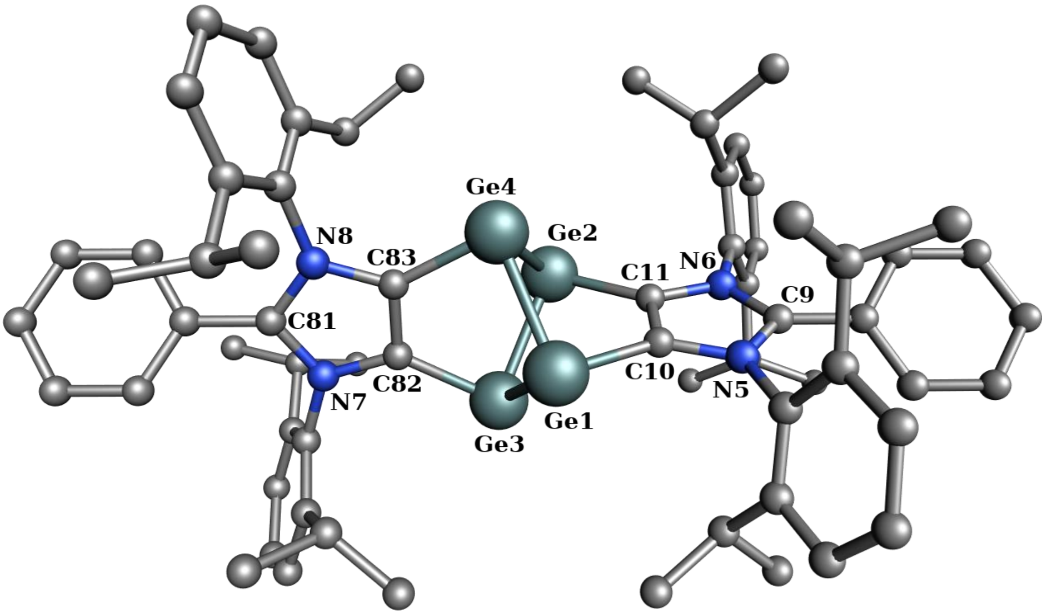

Figure S28. The optimized molecular structure of free **3** (C_2 symmetry) in the first triplet (**T**) electronic state. The hydrogen atoms are omitted for clarity. The internal working numeration of selected atoms is shown. Selected equilibrium parameters (Å, degrees) are: r(Ge3–Ge1) = 2.538, r(Ge3–Ge2) = 2.554, r(Ge4–Ge1) = 2.554, r(Ge4–Ge2) = 2.538, r(C9–N5) = 1.346, r(C9–N6) = 1.346, r(C10–Ge1) = 2.009, r(C10–N5) = 1.375, r(C11–Ge2) = 2.009, r(C11–N6) = 1.375, r(C11–C10) = 1.372, r(C81–N7) = 1.346, r(C81–N8) = 1.346, r(C82–Ge3) = 2.009, r(C82–N7) = 1.375, r(C83–Ge4) = 2.009, r(C83–N8) = 1.375, r(C83–C82) = 1.372, a(Ge1–Ge3–Ge2) = 82.3, a(Ge3–Ge1–Ge4) = 82.3, a(Ge3–Ge1–C10) = 89.2, a(Ge1–Ge3–C82) = 89.2, a(Ge3–Ge2–Ge4) = 82.2, a(Ge3–Ge2–C11) = 90.3, a(Ge2–Ge3–C82) = 90.3, a(Ge1–Ge4–Ge2) = 82.2, a(Ge4–Ge1–C10) = 90.3, a(Ge1–Ge4–C83) = 90.3, a(Ge4–Ge2–C11) = 89.1, a(Ge2–Ge4–C83) = 89.1, a(N5–C9–N6) = 106.0, a(C9–N5–C10) = 110.6, a(C9–N6–C11) = 110.6, a(Ge1–C10–N5) = 134.2, a(Ge1–C10–C11) = 119.4, a(N5–C10–C11) = 106.4, a(Ge2–C11–N6) = 134.1, a(Ge2–C11–C10) = 119.4, a(N6–C11–C10) = 106.4, a(N7–C81–N8) = 106.0, a(C81–N7–C82) = 110.6, a(C81–N8–C83) = 110.6, a(Ge3–C82–N7) = 134.2, a(Ge3–C82–C83) = 119.4, a(N7–C82–C83) = 106.4, a(Ge4–C83–N8) = 134.1, a(Ge4–C83–C82) = 119.4, a(N8–C83–C82) = 106.4, t(Ge4–Ge1–Ge3–Ge2) = 40.2, t(Ge1–Ge3–Ge2–Ge4) = –40.5, t(C10–Ge1–Ge3–Ge2) = –50.3, t(Ge1–Ge3–Ge2–C11) = 48.6, t(Ge3–Ge1–Ge4–Ge2) = –40.5, t(C82–Ge3–Ge1–Ge4) = –50.3, t(Ge3–Ge1–Ge4–C83) = 48.6, t(Ge3–Ge1–C10–N5) = –137.1, t(Ge3–Ge1–C10–C11) = 45.6, t(C82–Ge3–Ge1–C10) = –140.7, t(Ge1–Ge3–C82–N7) = –137.1, t(Ge1–Ge3–C82–C83) = 45.6, t(Ge3–Ge2–Ge4–Ge1) = 40.2, t(C82–Ge3–Ge2–Ge4) = 48.6, t(Ge3–Ge2–Ge4–C83) = –50.3, t(Ge3–Ge2–C11–N6) = 140.9, t(Ge3–Ge2–C11–C10) = –36.6, t(C82–Ge3–Ge2–C11) = 137.7, t(Ge2–Ge3–C82–N7) = 140.7, t(Ge2–Ge3–C82–C83) = –36.6, t(C10–Ge1–Ge4–Ge2) = 48.6, t(Ge1–Ge4–Ge2–C11) = –50.3, t(Ge4–Ge1–C10–N5) = 140.7, t(Ge4–Ge1–C10–C11) = –36.6, t(C83–Ge4–Ge1–C10) = 137.7, t(Ge1–Ge4–C83–N8) = 140.9, t(Ge1–Ge4–C83–C82) = –36.6, t(Ge4–Ge2–C11–N6) = –136.9, t(Ge4–Ge2–C11–C10) = 45.6, t(C83–Ge4–Ge2–C11) = –140.7, t(Ge2–Ge4–C83–N8) = –136.9, t(Ge2–Ge4–C83–C82) = 45.6, t(C10–N5–C9–N6) = –0.8, t(N5–C9–N6–C11) = –0.8, t(C9–N5–C10–Ge1) = –175.6, t(C9–N5–C10–C11) = 2.0, t(C9–N6–C11–Ge2) = –175.8, t(C9–N6–C11–C10) = 2.0, t(Ge1–C10–C11–Ge2) = –6.2, t(Ge1–C10–C11–N6) = 175.7, t(N5–C10–C11–Ge2) = 175.8, t(N5–C10–C11–N6) = –2.3, t(C82–N7–C81–N8) = –0.8, t(N7–C81–N8–C83) = –0.8, t(C81–N7–C82–Ge3) = –175.6, t(C81–N7–C82–C83) = 2.0, t(C81–N8–C83–Ge4) = –175.8, t(C81–N8–C83–C82) = 2.0, t(Ge3–C82–C83–Ge4) = –6.2, t(Ge3–C82–C83–N8) = 175.7, t(N7–C82–C83–Ge4) = 175.8, t(N7–C82–C83–N8) = –2.3.

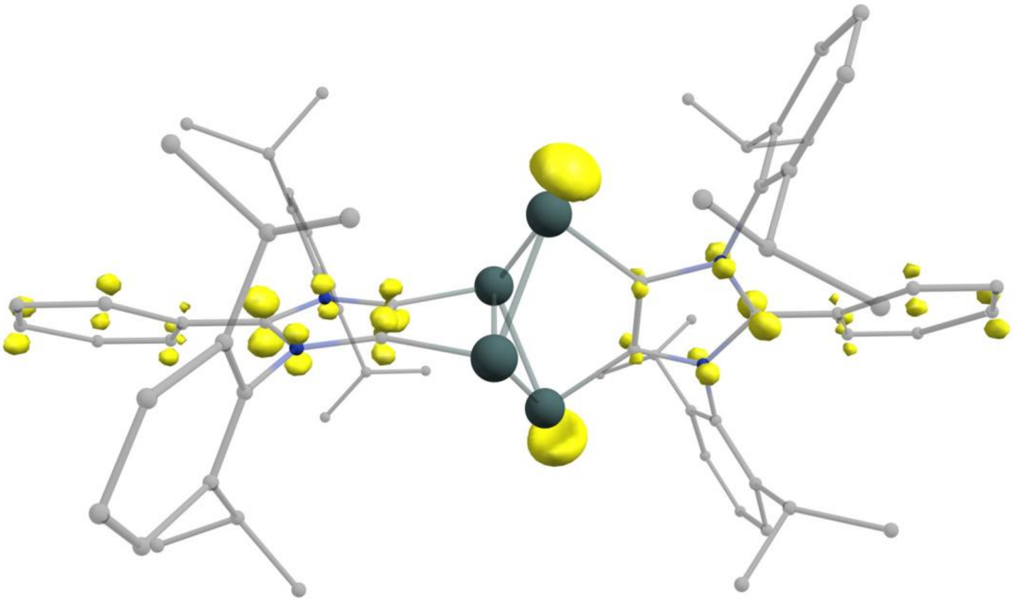

Figure S29. The FOD plot (isosurfaces 0.005 a.u. in yellow) of **3**. The Hydrogen atoms are omitted for clarity.

Table S8. The frontier molecular orbitals (isosurfaces 0.05 a.u.) and the corresponding energies (eV) in the TD-DFT calculation of **3**.

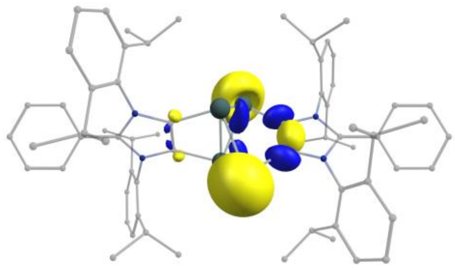 HOMO–5 (–6.7869)	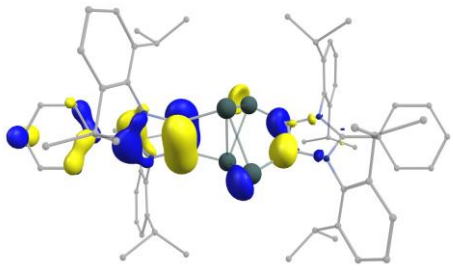 HOMO–4 (–6.3890)
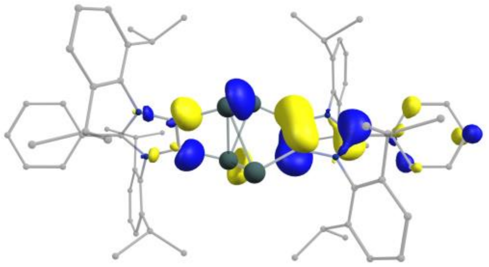 HOMO–3 (–5.9683)	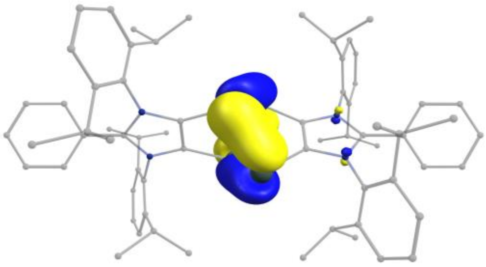 HOMO–2 (–5.3848)
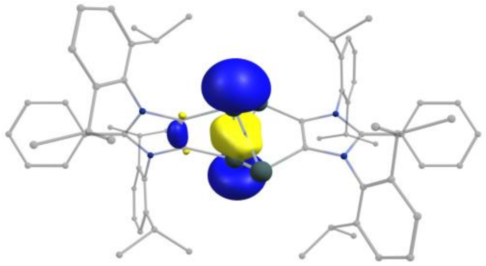 HOMO–1 (–5.2274)	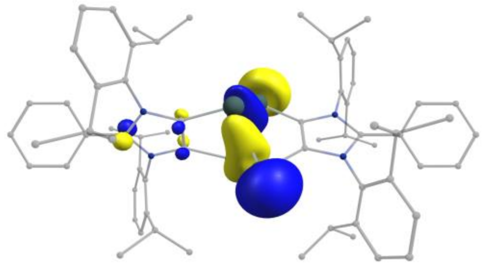 HOMO (–4.2840)
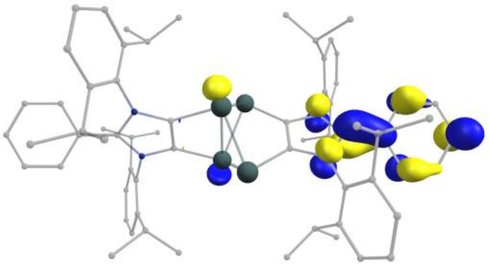 LUMO (–1.5340)	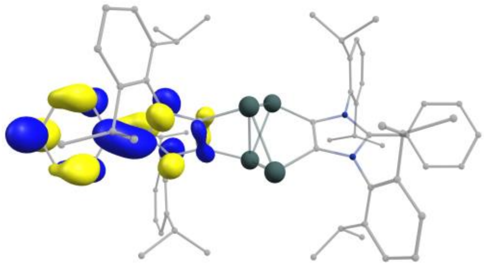 LUMO+1 (–1.3580)
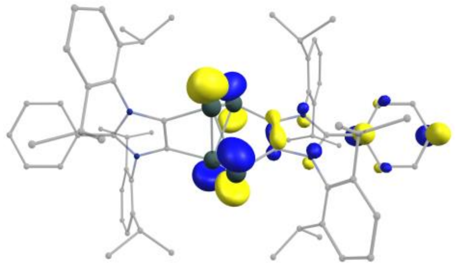 LUMO+2 (–0.9298)	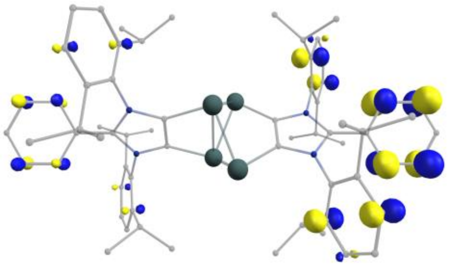 LUMO+3 (–0.6800)

Table S9. The most important transitions (vertical energy differences) in the TD-DFT calculation of **3**. The wavelengths λ in nm, the oscillator strengths f via the transition electric dipole moments and the assignments are listed.

λ, nm	f	Assignment
514	0.306	92 % HOMO → LUMO+1
332	0.414	57 % HOMO–3 → LUMO, 15 % HOMO → LUMO+15
308	0.417	37 % HOMO–2 → LUMO+3, 25 % HOMO–2 → LUMO+5, 16 % HOMO–3 → LUMO+1

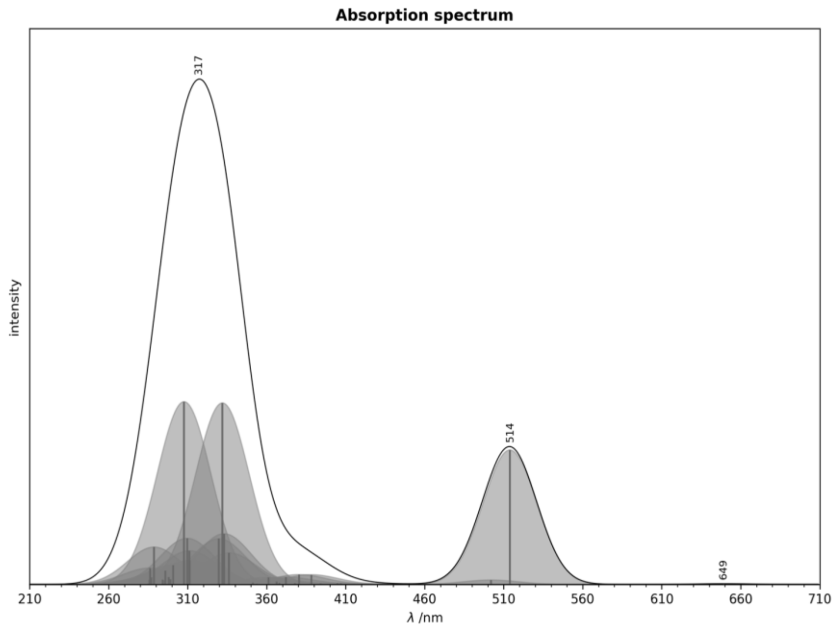

Figure S30. The simulated UV-Vis spectrum of **3** based on TD-DFT calculations. Individual transitions are shown as vertical sticks and are approximated as Gaussian functions (gray areas with 20 nm FWHM). The full line is the sum of all Gaussians. No empirical shift has been applied to the transition energies.

Table S10. The results of the NBO calculations for **3**. Natural charges and Wiberg bond indices are given for single atoms and atomic pairs, respectively.

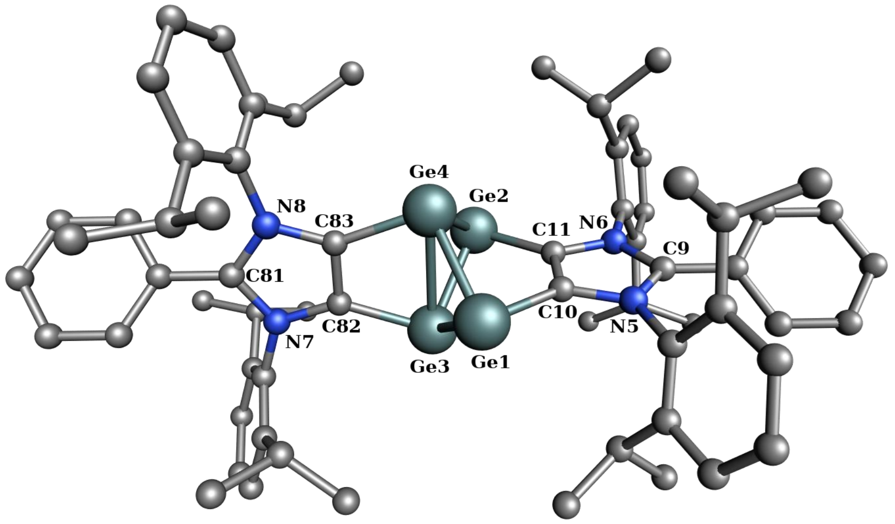

Atom or Atom Pair	Charge or Wiberg bond index
Ge1	0.10277
Ge2	0.10277
Ge3	0.15249
Ge4	0.15249
N5	–0.36545
N6	–0.36545
N7	–0.34251
N8	–0.34251
C9	0.37389
C10	–0.23846
C11	–0.23846
C81	0.35331
C82	–0.21112
C83	–0.21112
Ge1...Ge2	0.1688
Ge1–Ge3	0.7732
Ge1–Ge4	0.7607
Ge2–Ge3	0.7607
Ge2–Ge4	0.7732
Ge3–Ge4	0.5688
Ge1–C10	0.7510
Ge2–C11	0.7510
Ge3–C82	0.6939
Ge4–C83	0.6939
C10–C11	1.4524
C82–C83	1.5329

Table S11. The results of QTAIM calculations for **3**. Atom numbering is shown in Table S10.

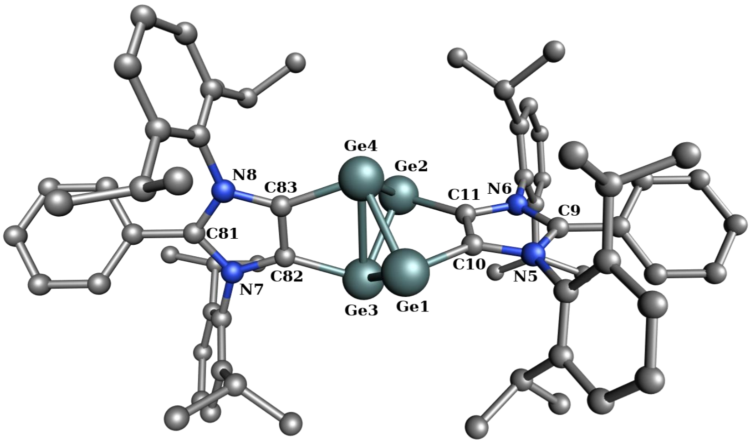

Atom or bond critical point	Charge, a.u.	Electron density, a.u.	Laplacian of electron density, a.u.	Bond ellipticity
Ge1	0.25			
Ge3	0.21			
C10	0.01			
C82	0.03			
Ge3–Ge4		0.048	0.011	13.8
Ge1–Ge3		0.066	–0.026	0.500
Ge1–C10		0.105	0.169	0.014
Ge3–C82		0.104	0.156	0.055
C10–C11		0.325	–0.949	0.216
C82–C83		0.345	–1.055	0.210

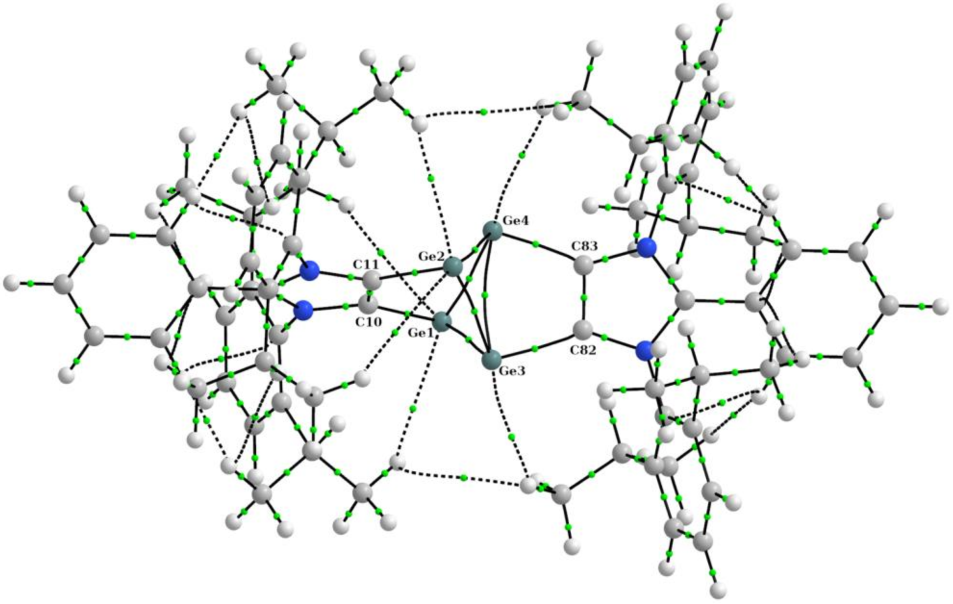

Figure S31. Molecular graph of **3** obtained from QTAIM analysis. Bond paths are shown as black solid and dashed lines. Green dots are bond critical points.

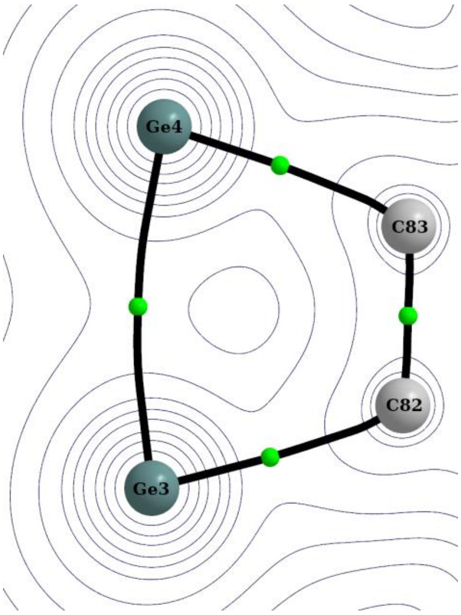

Figure S32. Contour plot of electron density in the Ge3–Ge4–C83–C82 plane of **3** obtained from QTAIM analysis. The bond paths are shown as black solid and dashed lines. The green dots are bond critical points.

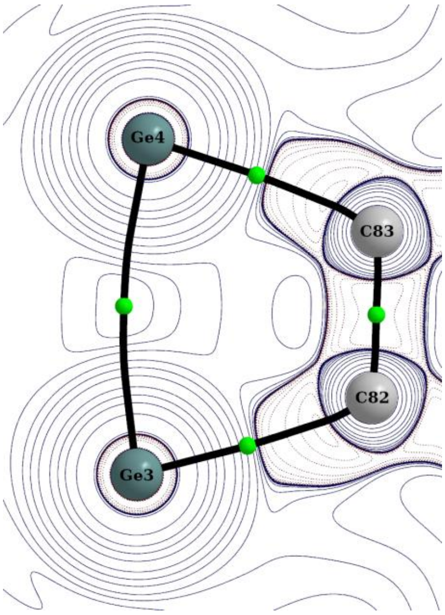

Figure S33. Contour plot of the Laplacian of electron density in the Ge3–Ge4–C83–C82 plane of **3** obtained from QTAIM analysis. Blue solid lines and red dotted lines correspond to positive and negative values, respectively.

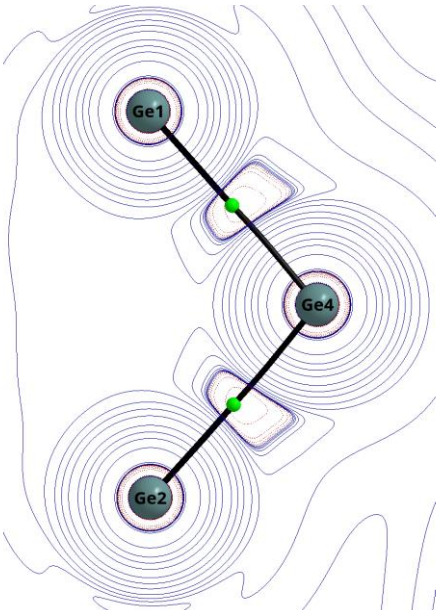

Figure S34. Contour plot of the Laplacian of electron density in the Ge1–Ge4–Ge2 plane of **3** obtained from QTAIM analysis. Blue solid lines and red dotted lines correspond to positive and negative values, respectively.

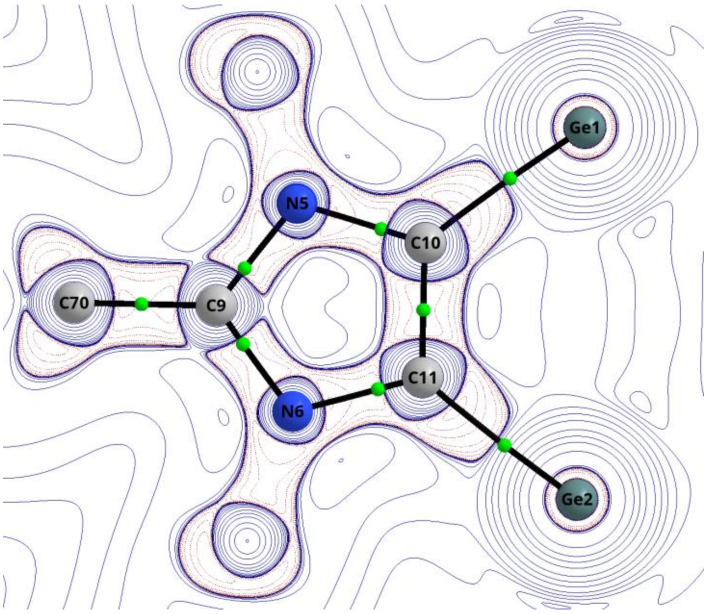

Figure S35. Contour plot of the Laplacian of electron density in the Ge1–C10–C11–Ge2 plane of **3** obtained from QTAIM analysis. Blue solid lines and red dotted lines correspond to positive and negative values, respectively.

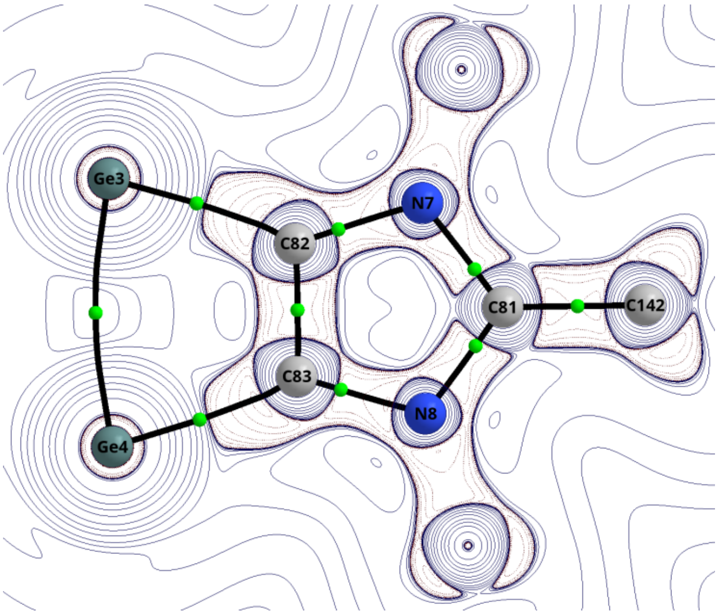

Figure S36. Contour plot of the Laplacian of electron density in the plane Ge3–C82–C83–Ge4 of **3** obtained from QTAIM analysis. Blue solid lines and red dotted lines correspond to positive and negative values, respectively.

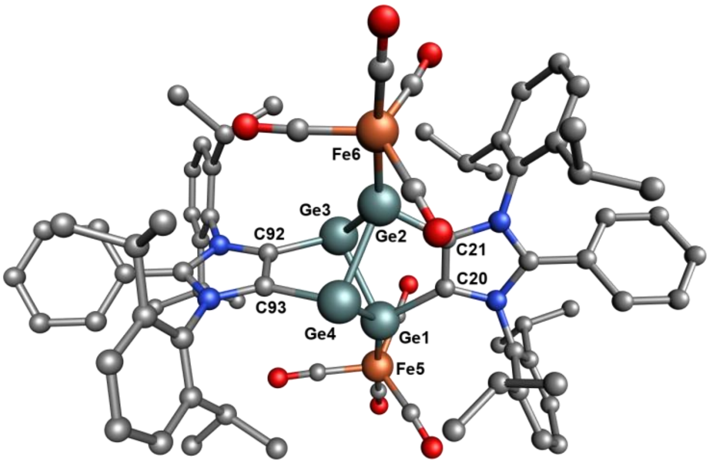

Figure S37. Optimized molecular structure of free **5** (C2 symmetry). Hydrogen atoms are omitted for clarity. Internal working numeration of selected atoms is shown. Selected equilibrium parameters (Å, degrees) are: r(Ge3-Ge1)=2.555, r(Ge3-Ge2)=2.448, r(Ge4-Ge1)=2.448, r(Ge4-Ge2)=2.555, r(Fe5-Ge1)=2.339, r(Fe6-Ge2)=2.339, r(C20-Ge1)=1.996, r(C21-Ge2)=1.996, r(C21-C20)=1.376, r(C92-Ge3)=2.000, r(C93-Ge4)=2.000, r(C93-C92)=1.356, a(Ge1-Ge3-Ge2)=88.9, a(Ge3-Ge1-Ge4)=71.0, a(Ge3-Ge1-Fe5)=118.8, a(Ge3-Ge1-C20)=89.2, a(Ge1-Ge3-C92)=98.7, a(Ge3-Ge2-Ge4)=71.0, a(Ge3-Ge2-Fe6)=146.0, a(Ge3-Ge2-C21)=87.5, a(Ge2-Ge3-C92)=99.2, a(Ge1-Ge4-Ge2)=88.9, a(Ge4-Ge1-Fe5)=146.0, a(Ge4-Ge1-C20)=87.5, a(Ge1-Ge4-C93)=99.2, a(Ge4-Ge2-Fe6)=118.8, a(Ge4-Ge2-C21)=89.2, a(Ge2-Ge4-C93)=98.7, a(Fe5-Ge1-C20)=123.2, a(Fe6-Ge2-C21)=123.2, a(Ge1-C20-C21)=121.5, a(Ge2-C21-C20)=121.5, a(Ge3-C92-C93)=112.7, a(Ge4-C93-C92)=112.7, t(Ge4-Ge1-Ge3-Ge2)=46.8, t(Ge1-Ge3-Ge2-Ge4)=-44.3, t(Fe5-Ge1-Ge3-Ge2)=-169.0, t(Ge1-Ge3-Ge2-Fe6)=-158.0, t(C20-Ge1-Ge3-Ge2)=-40.8, t(Ge1-Ge3-Ge2-C21)=45.7, t(Ge3-Ge1-Ge4-Ge2)=-44.3, t(C92-Ge3-Ge1-Ge4)=-52.4, t(Ge3-Ge1-Ge4-C93)=54.4, t(C92-Ge3-Ge1-Fe5)=91.8, t(Ge3-Ge1-C20-C21)=22.4, t(C92-Ge3-Ge1-C20)=-140.0, t(Ge1-Ge3-C92-C93)=39.7, t(Ge3-Ge2-Ge4-Ge1)=46.8, t(C92-Ge3-Ge2-Ge4)=54.4, t(Ge3-Ge2-Ge4-C93)=-52.4, t(C92-Ge3-Ge2-Fe6)=-59.3, t(Ge3-Ge2-C21-C20)=-48.6, t(C92-Ge3-Ge2-C21)=144.4, t(Ge2-Ge3-C92-C93)=-50.6, t(Fe5-Ge1-Ge4-Ge2)=-158.0, t(Ge1-Ge4-Ge2-Fe6)=-169.0, t(C20-Ge1-Ge4-Ge2)=45.7, t(Ge1-Ge4-Ge2-C21)=-40.8, t(C93-Ge4-Ge1-Fe5)=-59.3, t(Ge4-Ge1-C20-C21)=-48.6, t(C93-Ge4-Ge1-C20)=144.4, t(Ge1-Ge4-C93-C92)=-50.6, t(C93-Ge4-Ge2-Fe6)=91.8, t(Ge4-Ge2-C21-C20)=22.4, t(C93-Ge4-Ge2-C21)=-140.0, t(Ge2-Ge4-C93-C92)=39.7, t(Fe5-Ge1-C20-C21)=147.0, t(Fe6-Ge2-C21-C20)=147.0, t(Ge1-C20-C21-Ge2)=18.2, t(Ge3-C92-C93-Ge4)=5.1.

Table S12. The frontier molecular orbitals (isosurfaces 0.05 a.u.) and the corresponding energies (eV) in the TD-DFT calculation of **5**.

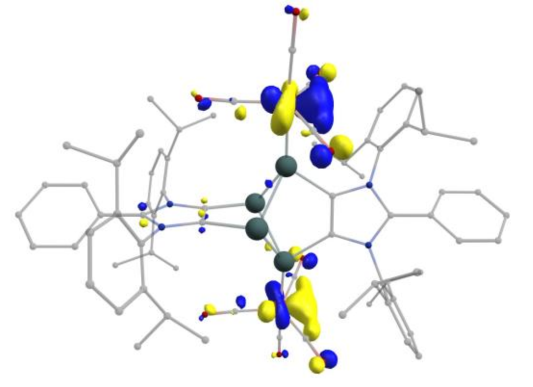	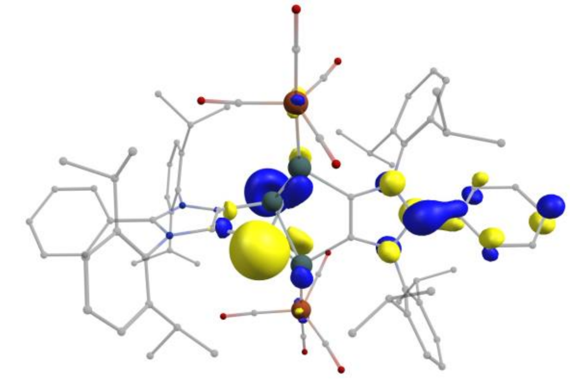
HOMO (–5.3863)	LUMO (–2.0744)

Table S13. Results of NBO calculations for **5**. Natural charges and Wiberg bond indices are given for single atoms and atomic pairs, respectively.

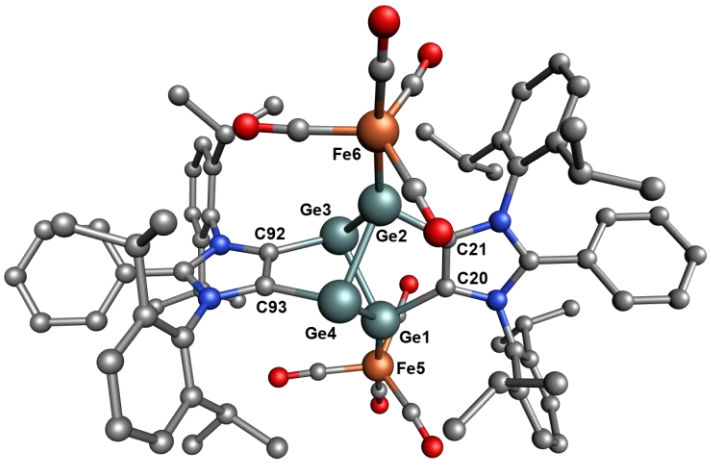

Atom or Atom Pair	Charge or Wiberg bond index
Ge1	0.34
Ge3	0.37
Fe5	–0.71
Ge1...Ge2	0.07
Ge1-Ge3	0.69
Ge1-Ge4	0.84
Ge3...Ge4	0.48
Ge1-Fe5	0.38

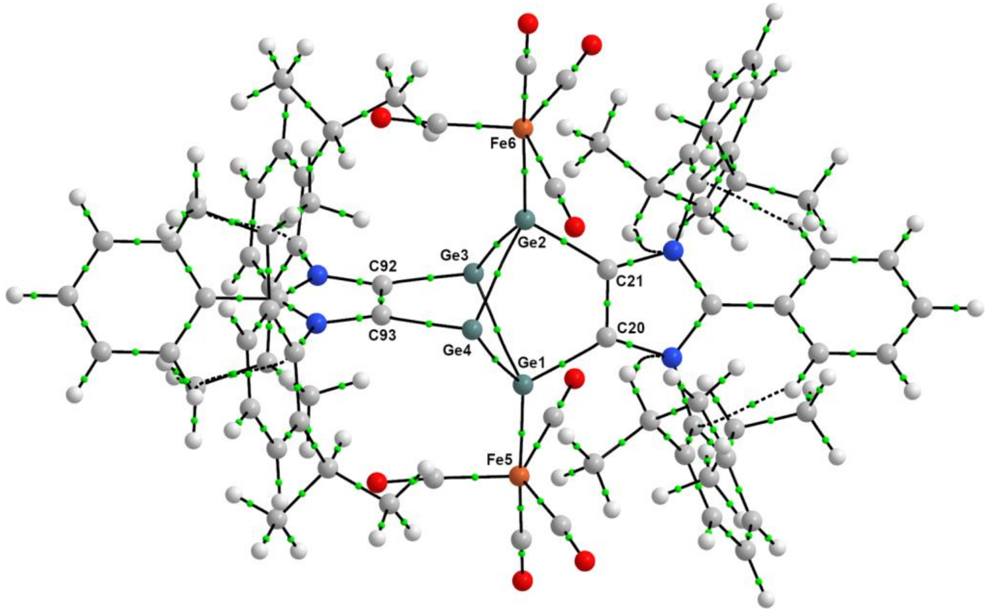

Figure S38. Molecular graph of **5** obtained in QTAIM analysis. Visualized are bond critical points (BCP, green dots) and respective bond paths (lines) if electron densities in BCPs were larger than 0.01 a.u. Weak bond paths are shown as dashed lines if the densities in corresponding BCPs were smaller than 0.025 a.u.

Table S14. The results of QTAIM calculations for **5**. Atom numbering is shown in Figure S38.

Atom or bond critical point	Charge, a.u.	Electron density, a.u.	Laplacian of electron density, a.u.	Bond ellipticity
Ge1	0.48			
Ge3	0.39			
C20	0.002			
C92	0.02			
Fe5	0.56			
Ge1–Ge3		0.065	–0.032	0.38
Ge1–Ge4		0.075	–0.047	0.32
Ge1–Fe5		0.073	0.056	0.03
Ge1–C20		0.112	0.162	0.06
Ge3–C92		0.110	0.153	0.08
C20–C21		0.331	–0.977	0.24
C92–C93		0.347	–1.061	0.23

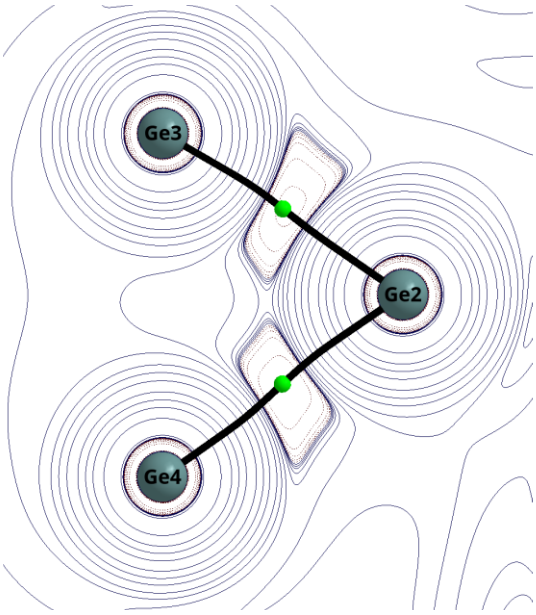

Figure S39. Contour plot of the Laplacian of electron density in the Ge3–Ge2–Ge4 plane of **5** obtained from QTAIM analysis. The bond paths are shown as black solid and dashed lines. The green dots are bond critical points.

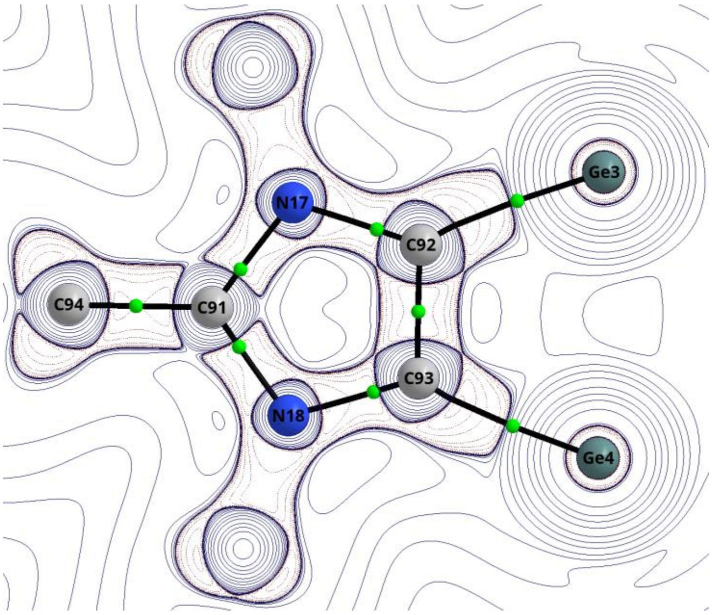

Figure S40. Contour plot of the Laplacian of electron density in the Ge3–C92–C93–Ge4 plane of **5** obtained from QTAIM analysis. Blue solid lines and red dotted lines correspond to positive and negative values, respectively.

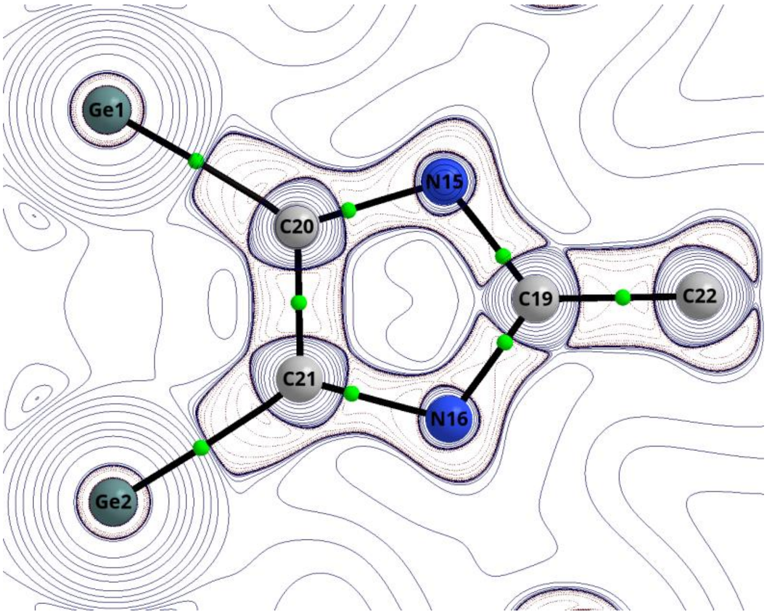

Figure S41. Contour plot of the Laplacian of electron density in the Ge1–C20–C21–Ge2 plane of **5** obtained from QTAIM analysis. Blue solid lines and red dotted lines correspond to positive and negative values, respectively.

References

[1] A. Merschel, Y. V. Vishnevskiy, B. Neumann, H. G. Stammler, R. S. Ghadwal, *Chem. Eur. J.* **2024**, *30*, e202303652.

[2] G. R. Fulmer, A. J. M. Miller, N. H. Sherden, H. E. Gottlieb, A. Nudelman, B. M. Stoltz, J. E. Bercaw, K. I. Goldberg, *Organometallics* **2010**, *29*, 2176–2179.

[3] O. V. Dolomanov, L. J. Bourhis, R. J. Gildea, J. A. K. Howard, H. Puschmann, *J. Appl. Cryst.* **2009**, *42*, 339–341.

[4] G. Sheldrick, *Acta Cryst. A* **2015**, *71*, 3–8.

[5] G. Sheldrick, *Acta Cryst. C* **2015**, *71*, 3–8.

[6] S. Grimme, A. Hansen, *Angew. Chem. Int. Ed.* **2015**, *54*, 12308–12313.

[7] F. Neese, *WIREs Comp. Mol. Sci.* **2022**, *12*, e1606.

[8] F. Neese, F. Wennmohs, A. Hansen, U. Becker, *Chem. Phys.* **2009**, *356*, 98–109.

[9] C. A. Bauer, A. Hansen, S. Grimme, *Chem. Eur. J.* **2017**, *23*, 6150–6164.

[10] a) F. Weigend, R. Ahlrichs, *Phys. Chem. Chem. Phys.* **2005**, *7*, 3297–3305; b) C. Adamo, V. Barone, *J. Chem. Phys.* **1999**, *110*, 6158–6170.

[11] a) E. Miliordos, K. Ruedenberg, S. S. Xantheas, *Angew. Chem. Int. Ed.* **2013**, *52*, 5736–5739; b) W. D. Laidig, H. F. Schaefer III, *J. Chem. Phys.* **1981**, *74*, 3411–3414.

[12] Y. Guo, K. Sivalingam, E. F. Valeev, F. Neese, *J. Chem. Phys.* **2016**, *144*, 094111.

[13] C. R. Landis, F. Weinhold, *Valency and Bonding: A Natural Bond Orbital Donor-Acceptor Perspective*, Cambridge University Press, Cambridge, **2005**.

[14] E. D. Glendening, J. K. Badenhoop, A. E. Reed, J. E. Carpenter, J. A. Bohmann, C. M. Morales, P. Karafiloglou, C. R. Landis, F. Weinhold, *NBO 7.0*, Theoretical Chemistry Institute, University of Wisconsin, Madison, **2018**.

[15] R. F. W. Bader, *Atoms in Molecules: A Quantum Theory*, Oxford University Press, **1990**.

[16] K. A. Todd, AIMAII (Version 19.10.12), https://aim.tkgristmill.com/ ed., **2019**.
